# Characterization of a unique catechol-*O*-methyltransferase as a molecular drug target in parasitic filarial nematodes

**DOI:** 10.1371/journal.pntd.0012473

**Published:** 2024-08-30

**Authors:** Md Mukthar Mia, Idrees Mehraj Allaie, Xuejin Zhang, Kun Li, Shahbaz M. Khan, Saki Kadotani, William H. Witola

**Affiliations:** 1 Department of Pathobiology, College of Veterinary Medicine, University of Illinois Urbana-Champaign, Urbana, Illinois, United States of America; 2 Institute of Traditional Chinese Veterinary Medicine, MOE Joint International Research Laboratory of Animal Health and Food Safety, College of Veterinary Medicine, Nanjing Agricultural University, Nanjing, China; 3 Department of Veterinary Clinical Medicine, College of Veterinary Medicine, University of Illinois Urbana-Champaign, Urbana, Illinois, United States of America; Universidad de la República Uruguay: Universidad de la Republica Uruguay, URUGUAY

## Abstract

**Background:**

Filarial nematodes cause severe illnesses in humans and canines including limb deformities and disfigurement, heart failure, blindness, and death, among others. There are no vaccines, and current drugs against filarial nematodes infections have only modest effects and are prone to complications.

**Methodology/principal findings:**

We identified a gene (herein called DiMT) encoding an *S*-adenosyl-*L*-methionine (SAM)-dependent methyltransferase with orthologs in parasite filarial worms but not in mammals. By *in silico* analysis, DiMT possesses catalytic sites for binding SAM and catecholamines with high affinity. We expressed and purified recombinant DiMT protein and used it as an enzyme in a series of SAM-dependent methylation assays. DiMT acted specifically as a catechol-*O*-methyltransferase (COMT), catalyzing catabolic methylation of dopamine, and depicted Michaelis Menten kinetics on substrate and co-substrate. Among a set of SAM-dependent methyltransferase inhibitors, we identified compounds that bound with high affinity to DiMT’s catalytic sites and inhibited its enzymatic activity. By testing the efficacy of DiMT inhibitors against microfilariae of *Dirofilaria immitis* in culture, we identified three inhibitors with concentration- and time-dependent effect of killing *D*. *immitis* microfilariae. Importantly, RNAi silencing of a DiMT ortholog in *Caenorhabditis elegans* has been shown to be lethal, likely as a result of excessive accumulation of active catecholamines that inhibit worm locomotion, pharyngeal pumping and fecundity.

**Conclusions/significance:**

Together, we have unveiled DiMT as an essential COMT that is conserved in parasitic filarial nematodes, but is significantly different from mammalian COMTs and, therefore, is a viable target for development of novel drugs against filarial nematode infections.

## Author summary

Parasitic filarial nematodes cause serious illnesses in humans and canines including limb deformities and disfigurement, blindness, heart failure, and death, among others. There are no vaccines, and current drugs in use against filarial nematodes infections have only modest effects and are prone to complications. In this study, we explored a unique enzyme (DiMT), conserved in all parasitic filarial nematodes but not in mammalian species. Using computation, biochemistry and molecular biology approaches, we validated DiMT as a catechol-*O*-methyltransferase that functions to inactivate catecholamines to prevent their accumulation and hence block their deleterious effects in filarial nematodes, including loss of worm viability, reproduction and survival. Subsequently, we identified DiMT’s chemical inhibitors and demonstrated that they possess anti-filarial nematode efficacy through blocking the inactivation of catecholamines. Together, we have unveiled DiMT as a viable molecular target for the development of new drugs for treating filarial nematode infections.

## 1. Introduction

*S*-adenosyl-*L*-methionine (SAM)-dependent methyltransferases catalyze methyl transfer reactions [1,2]. There are several structurally distinct families of SAM-dependent methyltransferases, with each family representing a series of enzymes with structurally similar active sites [2]. Methylation is integral to maintenance of life. For instance, DNA methylation is a prerequisite for gene expression and mutation repair, and subsequent post-translational methylation of expressed proteins modifies their functional activity. Methylation reactions also play important roles in the metabolic inactivation of many molecules that contain an amino functionality [1]. Examples of some well characterized SAM-dependent methyltransferases include: Catechol-*O*-methyltransferase (COMT) which catalyzes the methylation of catecholamine neurotransmitters leading to their inactivation [3,4]; Histamine *N*-methyltransferase that catalyzes inactivation of histamine through methylation [5]; Thiol methyltransferase that detoxifies gut-derived xenobiotics through *S*-methylation [6]; and Thiopurine methyltransferase that catalyzes *S*-methylation of thiopurine, thiopyrimidines and thiophenols leading to their inactivation [7].

Catecholamines, including dopamine and serotonin, have been shown to be expressed in the nematode *Caenorhabditis elegans* in which they function as neurotransmitters or neuromodulators [7]. However, elevated activity of catecholamines has been shown to inhibit *C*. *elegans*’ locomotion, pharyngeal pumping and fecundity [7–11]. Because locomotion and pharyngeal pumping are essential activities for the survival of nematode worms, while fecundity is critical for production of worm progeny, catecholamines in nematodes are considered as important modulators of worm activity and survival [12]. Catecholamines are degraded by methylation catalyzed by COMTs [13–15]. Therefore, the methylation of catecholamines in nematodes would serve to abrogate their deleterious activities in the worms.

Herein, we describe the functional characterization of a unique *Dirofilaria immitis* COMT (DiMT) that has conserved orthologs in other parasitic filarial nematodes, but not in mammals. Filarial nematodes, including *D*. *immitis* (causes heart failure in canines), *Brugia malayi* and *Wuchereria bancrofti* (agents for lymphatic filariasis in humans), *Onchocerca volvulus* (cause of river blindness in humans) and *Loa loa* (human eyeworm), are vector-borne parasitic filarial nematodes. There are no effective vaccines, and current drugs in use against filarial nematodes have only modest effect and are prone to complications. Therefore, in this study, we endeavored to explore the inhibition of the molecular functional activity of a highly conserved unique filarial nematode COMT, and to explore the implication of its chemical inhibitors for development of novel therapeutics against filarial nematodes infections.

## 2. Materials and methods

### 2.1. Ethics statement

All experiments involving the use of animals in this study were approved by the University of Illinois Urbana-Champaign Institutional Animal Care and Use Committee under protocol number 21144.

### 2.2. DiMT gene identification, analysis and phylogeny construction

We used the *D*. *immitis* genome data [16] for predicted proteomes possessing orthologs in *C*. *elegans* with RNAi phenotypes that are lethal, lack significant BLAST matches (*E-*value ≤ 10^−5^) in *Homo sapiens*, and have predicted functions as enzymes. Based on those criteria, the DiMT gene coding sequence was retrieved from the UniprotKB database (https://www.uniprot.org/uniparc?query=nDi.2.2.2.t06229) with transcript identification number nDi.2.2.2.t06229. To identify the sequence similarity, BLASTP (http://blast.ncbi.nlm.nih.gov/Blast.cgi) was performed using DiMT as the query sequence. NCBI CDD [17] and the InterProScan v.5.8–49.0 server [18] were used to determine the functional domain and motif of the DiMT protein. To identify the conserved regions, sequence alignments of different SAM-dependent methyltransferases (SDMTs) proteins’ domains were generated using ClutalW. The phylogenetic analysis was performed using MEGA X version 6.0 followed by phylogenetic reconstructions by the neighbor-joining methods (Parameters: 500 bootstrap replications). Trees were thereafter manually labeled using the MEGA view option. While MEGA X version 6.0 is considered an older version, it has advantages in that it is user friendly and accurately estimates the relationship between different molecular sequences. Further, it supports time tree inference by utilizing the RelTime method to estimate divergence times across all phylogenetic nodes. The Time tree Wizard offers a user-friendly interface for configuring phylogeny and calibration restrictions. Additionally, MEGA’s ability to handle sequence data sets has increased because of enhanced memory management and algorithms.

### 2.3. Model prediction for DiMT protein

Prediction of unknown protein structures is crucial in drug development as it can facilitate the identification of small molecules that can fit into a target protein’s binding pocket and generate favorable interactions. In the absence of a solved 3-dimensional (3D) structure of DiMT protein, the AlphaFold2 (AF2) protein prediction tool (https://github.com/deepmind/alphafold/AlphaFold.ipynb) was used to predict the 3D structure of DiMT protein. The model can predict the structure with precision comparable to experimentally solved structures using techniques such as cryo-EM, NMR, and X-ray crystallography [19]. The coding sequence of the DiMT protein was retrieved from the UniParc database (nDi.2.2.2.t06629) and used as input in the AF2 software. The AF2 software generates a multiple sequence alignment (MSA) by querying various protein sequence databases with the input amino acid sequence, yielding a per-residue local distance difference test (pLDDT) confidence score [20]. The accuracy of the AF2 predicted structure was further evaluated using a Ramachandran plot from PDBsum with the help of a PROCHECK server [21]. Furthermore, binding site amino acid residues of DiMT protein were predicted using the COACH server [22]. For *in silico* dock simulations, catecholamine (dopamine) and non-catecholamines (octopamine, tyramine, histamine, 2-mercaptoethanol and phosphoethanolamine) were used as ligands. For prediction of co-substrate, *S*-adenosyl-*L*-methionine (SAM) was used as ligand. A blind docking simulation using AutoDock Vina [23] was performed to determine the amino acid residues to which the ligands bound. The grid box was set as 64×64×64 Å with -5.333, -1.306, and 18.333 (x, y, z) dimensions. The receptor-ligand interactions were visualized using Discovery Studio 3.0 [24].

### 2.4. Molecular docking of inhibitors on DiMT protein

Inhibitor binding affinities and stability at the catalytic sites of the DiMT protein were determined by *in silico* docking using Autodock Vina (Scripps Institute, USA). PubChem database (https://pubchem.ncbi.nlm.nih.gov/) was used to obtain the inhibitors’ 3D structures that were used as ligands for docking using PyMoL and Autodock MGL tools [25]. The predicted DiMT protein structure was prepared as a macromolecule and saved in PDBQT file format. To facilitate the docking, the DiMT protein structure was placed in the Autodock MGL tool, and the water molecules were eliminated. Hydrogen bond interactions in the receptor-ligand complex were elaborated. Finally, Kollman charges were added to exhibit the electrostatic interactions between receptor and ligand. To evaluate the rotatable chemical bonds in the ligands, we examined the PubChem database, which offers extensive chemical data with accurate numbers of rotatable bonds in the ligands [26,27]. We discovered 4 rotatable bonds for each of NSC177383 and NSC56140, while NSC145612 had 5 rotatable bonds. The AutoDock Vina program can successfully dock ligands with an average of 7.30 rotatable bonds or even more [28]. We conducted three "Blind Docking" runs using semi-flexible docking with a rigid DiMT protein and flexible ligands. During each run, all rotatable bonds remained unbound to ensure unbiased outcomes. Blind docking with a grid box (64×64×64 Å) and the active site center set as -5.333, -1.306, and 18.333 (x, y, z), with a value of 10 for exhaustiveness was employed. Autodock vina uses an exhaustiveness of 8 (the maximum default value), which we extended to 10 in order to determine if it would create different poses in comparison to top-ranked poses. Discovery Studio 3.0 was used to visualize and analyze the receptor-ligand interactions [29]. Using PyMoL v2.5.5, we checked every docking pose to see whether rotatable chemical bonds took part in the formation of hydrogen bonds.

### 2.5. Cloning, expression, and purification of putative DiMT protein

The coding sequence of the DiMT protein (Accession number nDi.2.2.2.t06229) was synthesized by Integrated DNA Technologies (IDT, USA) and supplied cloned in pUCIDT (AMP) vector. Before use, the lyophilized vector was reconstituted in sterile molecular-grade nuclease-free water. The following primers were used for PCR amplification of the coding sequence of DiMT for directional cloning in the pPink-HC expression vector (PichiaPink Expression System, Invitrogen): 5′-*GAATTC*GCC**ATG**ATTGGTTATTTAATGGATATTTTAGATCGACT-3′ (Forward primer with the *EcoRI* restriction site italicized, Kozak sequence underlined, and start codon in bold) and 5′-*GGTACC***TTA**ATGATGATGATGATGATGATCAAGCTCTTTGATTC-3′ (Reverse primer with the *KpnI* restriction site italicized, stop codon in bold, and the added C-terminal His-tag underlined). The recombinant pPink-HC expression vector was sequenced to confirm identity of the DiMT insert followed by transformation into PichiaPink Strain-1 expression yeast (*Pichia pastoris*). The transformed PichiaPink were grown on Pichia Adenine Dropout (PAD) agar (Invitrogen) for selecting transformants at 30°C for 3 days. Positive transformant colonies were confirmed by colony PCR using a combination of insert- and vector-specific primers. The positive white colonies were propagated and cryopreserved for protein expression.

For protein expression, transformed PichiaPink was cultured for 2–3 days (OD_600_ of ~4) in 25 mL of MGY medium (1% glycerol; 1.34% YNB, and 0.00004% biotin) at 28°C with shaking. The 25 mL culture was inoculated in 300 mL of fresh MGY medium and cultured for 3 days at 28°C with shaking. Yeast cells were harvested by centrifugation and pellets resuspended in fresh 200 mL MM medium (1.34% YNB; 0.00004% and biotin) containing 0.5% methanol for induction of protein expression. The yeast was cultured at 28°C with shaking and supplementation with 0.5% methanol every 24 h for a total of 3 days. Yeast cells were harvested by centrifugation and pellets stored at -80°C for protein purification. To purify the recombinant DiMT protein, yeast pellets from **-**80°C were thawed on ice and resuspended in lysis buffer (300 mM NaCl; 50 mM NaH_2_PO_4,_ and 10 mM Imidazole, pH 8.0) supplemented with EDTA-free protease inhibitor cocktail (Thermo Scientific), 600 units of benzonase (EMD Millipore), and 30 kU of lysozyme and incubated for 30 minutes on ice. The suspension was sonicated to lyse the cells and the supernatant collected by centrifugation at 20,000 rpm for 20 minutes at 4°C. Pre-washed 2 ml of Ni-NTA His-bind resin suspension (Novagen) was added to 10 ml of yeast cell extract supernatant and incubated for 2 h at 4°C with agitation. The His-tagged recombinant proteins were purified under native conditions by nickel-affinity chromatography following the resin manufacturer’s instructions (Novagen). The eluted protein was dialyzed overnight using a dialysis buffer containing 10 mM Hepes-KOH (pH 7.8) and 1 mM dithiothreitol. The integrity and purity of the protein was determined using SDS-PAGE. A Qubit 3.0 fluorometer (Life Technologies) was used to quantify protein concentration.

### 2.6. Enzyme activity and kinetics assays

To determine the enzymatic activity and kinetics, a commercially available MTase-Glo methyltransferase activity assay kit (Promega) was customized for use with DiMT protein as enzyme, SAM as methyl-donor, and dopamine as substrate. Initially, enzyme activity of DiMT was determined using a reaction mixture containing 1.5 μL SAM (final concentration of 30 μM), 1.8 μL dopamine (final concentration 360 μM), and varied concentrations of DiMT purified protein. For all enzymatic experiments, the dialysis buffer was used to make up the final reaction volume to 50 μL. To determine the enzyme kinetics on SAM, a fixed concentration of optimized DiMT protein concentration (90 ng/μL) and dopamine as substrate (360 μM) were used with varying concentrations of SAM (0 to 60 μM). Likewise, to evaluate the enzyme kinetics on dopamine as a substrate, fixed concentrations of DiMT purified protein (90 ng/μL) and SAM (40 μM) were utilized, with varying concentrations of dopamine (0 to 600 μM). A negative control reaction without DiMT protein, but with an equal volume of dialysis buffer was included. The reactions were performed in a flat-bottomed opaque white 96 well plate and incubated at 23°C for 30 minutes in the dark, following which 12.5 μL of 5x MTase-Glo Reagent was added to each reaction and incubated for a further 30 minutes. Next, 62.5 μL of MTase-Glo Detection Solution was added to each reaction and the plate incubated for 30 minutes at 23°C in the dark. Luminescence was read using a SpectraMax ID5 Microplate Reader (Molecular Devices, United States). Sample readouts were normalized with the negative control. GraphPAD PRISM v9 software was used to fit the Michaelis-Menten model to the substrate-velocity data to determine the enzymatic kinetic parameters of DiMT. To determine DiMT’s substrate specificity, non-catecholamines (octopamine, tyramine, histamine, 2-mercaptoethanol and phosphoethanolamine) were used as substrates in the assay using fixed concentration of DiMT (90 ng/μL), SAM (40 μM) and substrate (300 μM).

### 2.7. Identification of inhibitors for enzymatic activity of DiMT

To identify inhibitors for DiMT enzymatic activity, an optimized small library of seven natural compounds with inhibitory activity against methyltransferases reported previously [30] was used. The compounds were from the Natural Products Set IV obtained from the National Cancer Institute/Developmental Therapeutics Program Open Chemical Repository. The compounds were reconstituted in DMSO. The test MTase-Glo reaction mixtures consisted of 50 μM test compounds, 40 μM SAM, 400 μM substrate (dopamine), and 90 ng/μL of DiMT protein, with the final volume made up to 50 μL with protein dialysis buffer. The volume of DMSO (test compound solvent) in the reactions was limited to less than 1% (v/v) of the total reaction volume. Positive control reaction mixtures did not contain a test compound but contained an equal volume of DMSO. For negative control reactions, instead of DiMT protein, an equal volume of dialysis buffer was added to the reaction mixture. The MTase-Glo reactions were carried out exactly as stated above. The mean percent inhibition (MPI) of the activity of DiMT by the compounds was derived using the following formula:

MPI = [(ΔRLU_positive_− ΔRLU _compound_) / ΔRLU _positive_] ×100

Where:

- ΔRLU _positive_ is the relative luminescence units (RLU) for the positive control reaction minus that of the negative control reaction RLU
- ΔRLU_compound_ the relative RLU value for the reaction with test compound minus the negative control reaction RLU.

Compounds that showed inhibitory activity against DiMT at the initial 50 μM were further tested at varying concentrations and their half maximal inhibitory concentration (IC_50_) values derived by applying the non-linear regression analysis curve fit to the mean dose-response data for varying concentrations of each compound using GrahPad PRISM v9.

### 2.8. Analysis of the effect of DiMT inhibitors against microfilariae of *D*. *immitis*

Candidate inhibitors were tested for *in vitro* efficacy against live *D*. *immitis* microfilariae. Live microfilariae were extracted from freshly drawn heparinized blood from a naturally infected dog diagnosed by the Teaching Animal Hospital at the College of Veterinary Medicine, University of Illinois Urbana-Champaign, USA. Approximately 10 ml of heparinized blood was drawn from the external jugular vein and maintained at 4°C until use. To confirm the presence of *D*. *immitis* microfilariae in the blood, 20 μL of the blood was used to make a wet smear that was then immediately examined under a light microscope using a 10x objective. To purify the live microfilariae, 2 ml of blood was diluted at a ratio of 1:10 with 1x eBioscience RBC Lysis Buffer (Multi-species; Thermo Fisher Scientific) and mixed by inverting the tube three times. The mixture was incubated at room temperature for 15 minutes followed by centrifugation at 350 x*g* at room temperature for 10 minutes. The supernatant was decanted, and the pellet (microfilariae) was resuspended in 20 ml of PBS. After mixing, the suspension was centrifuged at 300 x*g* for 10 minutes, the supernatant decanted and the microfilariae resuspended in pre-warmed RPMI medium supplemented with 10% fetal calf serum, 2.05 mM l-glutamine, 1 mM sodium pyruvate, 1.5 g/L sodium bicarbonate, 1% (v/v) penicillin-streptomycin-amphotericin B (Fungizone) (Life Technologies). The microfilariae were seeded at 100 worms per well in 0.5 ml of medium in 24-well plates. For each test inhibitor, three sets of microfilariae cultures were maintained as follows: Negative control wells without inhibitor but containing a volume (≤ 2% v/v) of DMSO equivalent to that contained in the solution of the reconstituted inhibitor to be tested. Positive control wells were treated with ivermectin (Sigma-Aldrich) at varying concentrations as a standard drug. Test wells were treated with varying concentrations of the test inhibitors. The cultures were incubated at 37°C with 5% CO_2_. At time intervals of 24, 48, 72, 96, and 120 h after start of treatment of the cultures, the percentage of completely immotile (dead) microfilariae was determined in each well using an inverted phase contrast light microscope with a heated base. The percentage values of the completely immotile parasites at varying concentrations of the test inhibitor relative to the DMSO-treated cultures were used to derive the antiparasitic half-minimal effective concentrations (EC_50_) using nonlinear regression analysis by GraphPad Prism software version 9.0 program. To determine if immotile microfilariae in the cultures were dead, at 120 h post-treatment with compounds, the microfilariae were washed 4 times with fresh medium without compound. Briefly, the cultures were transferred to 15 ml conical tubes and centrifuged to pellet the microfilariae. The supernatant was discarded, and the microfilariae resuspended in fresh medium without compound. The wash process was repeated 3 times and the microfilariae were re-seeded in 24 well plates and cultured for a further 72 h with observation every 12 h to determine if they show any sign of motility.

### 2.9. Statistical analyses

All *in vitro* experiments were performed using 3 technical replicates, and all assays were repeated 3 times using independent biological samples. All statistical analyses were performed using GraphPad PRISM v9. Normality of data distribution was assessed using Q-Q normal probability plots and the Shapiro-Wilk normality test. Statistical comparisons between the treatment groups and the vehicle control group were done by a non-parametric Kruskal-Wallis test or a parametric one-way analysis of variance (ANOVA) test with the Dunn’s or the Dunnett’s multiple comparison *post hoc* tests respectively, as appropriate for the data. *P* values of 0.05 or less were considered significant.

## 3. Results

### 3.1. DiMT coding sequence analysis and phylogeny

**Fig 1:**
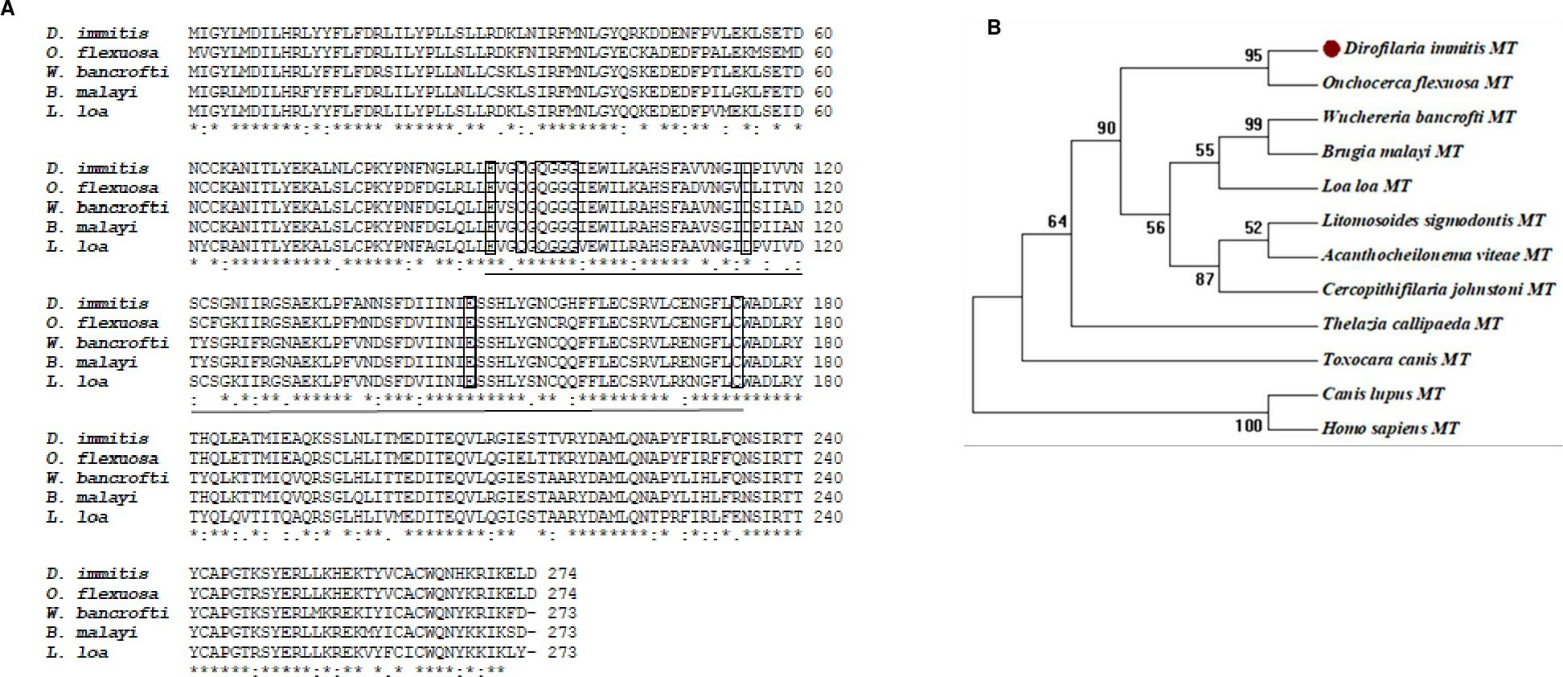
Analysis of *Dirofilaria immitis* putative catechol-*O*-methyltransferase (DiMT) amino acid sequence. **(A)** Multiple sequence alignment of DiMT protein with its orthologs in other filarial nematodes including *Brugia malayi* (XP_001898718.1), *Onchocerca flexuosa* (0ZCO7346.1), *Loa loa* (XP_003138136.1), and *Wuchereria bancrofti* (VDM10472.1). The catecholamine substrate-binding amino acid residues are boxed, while the *S*-adenosyl-L-methionine catalytic site, that is a conserved domain of the AdoMet_MTases superfamily of methyltransferases, is underlined. Asterisks (*) indicate positions with a single, fully conserved residue. **(B)** Phylogenetic construct showing that DiMT protein sequence and its orthologs in filarial nematodes (*B*. *malayi*, *O*. *fexuosa*, *L*. *loa* and *W*. *bancrofti*) are closely related while being distant from catechol-*O*-methyltransferases from mammalian species and other organisms. The phylogeny robustness was assessed using bootstrap resampling of 500 replicates and is depicted by nodal values.

The transcript sequence of DiMT from *D*. *immitis* retrieved from the UniprotKB database with identification number nDi.2.2.2.t06229 contained an 825 bp coding sequence that translated into a 275 amino acids protein sequence. BLAST search analysis revealed that the DiMT protein sequence has orthologs in other filarial nematodes including *Onchocerca flexuosa*, *Brugia malayi*, *Loa loa*, and *Wuchereria bancrofti* (with accession numbers 0ZCO7346.1, XP_001898718.1, XP_003138136.1 and VDM10472.1, respectively) that showed 88.32%, 81.92%, 81.55% and 81.55% sequence homology, respectively (Fig 1A). Corroboratively, phylogenetic analysis demonstrated that DiMT is closely related to its putative catechol-*O*-methyltransferase (COMT) orthologs in various filarial nematodes but is very distant from those in mammalian hosts, including humans and canines (Fig 1B). By homology comparison to mammalian COMTs, DiMT was only 23.6% and 12.2% similar to human COMT (Accession number A0A7I2V370) and canine COMT (Accession number A0A8C0Z488), respectively.

### 3.2. *In silico* modelling of DiMT protein depicts COMT configuration

**Fig 2:**
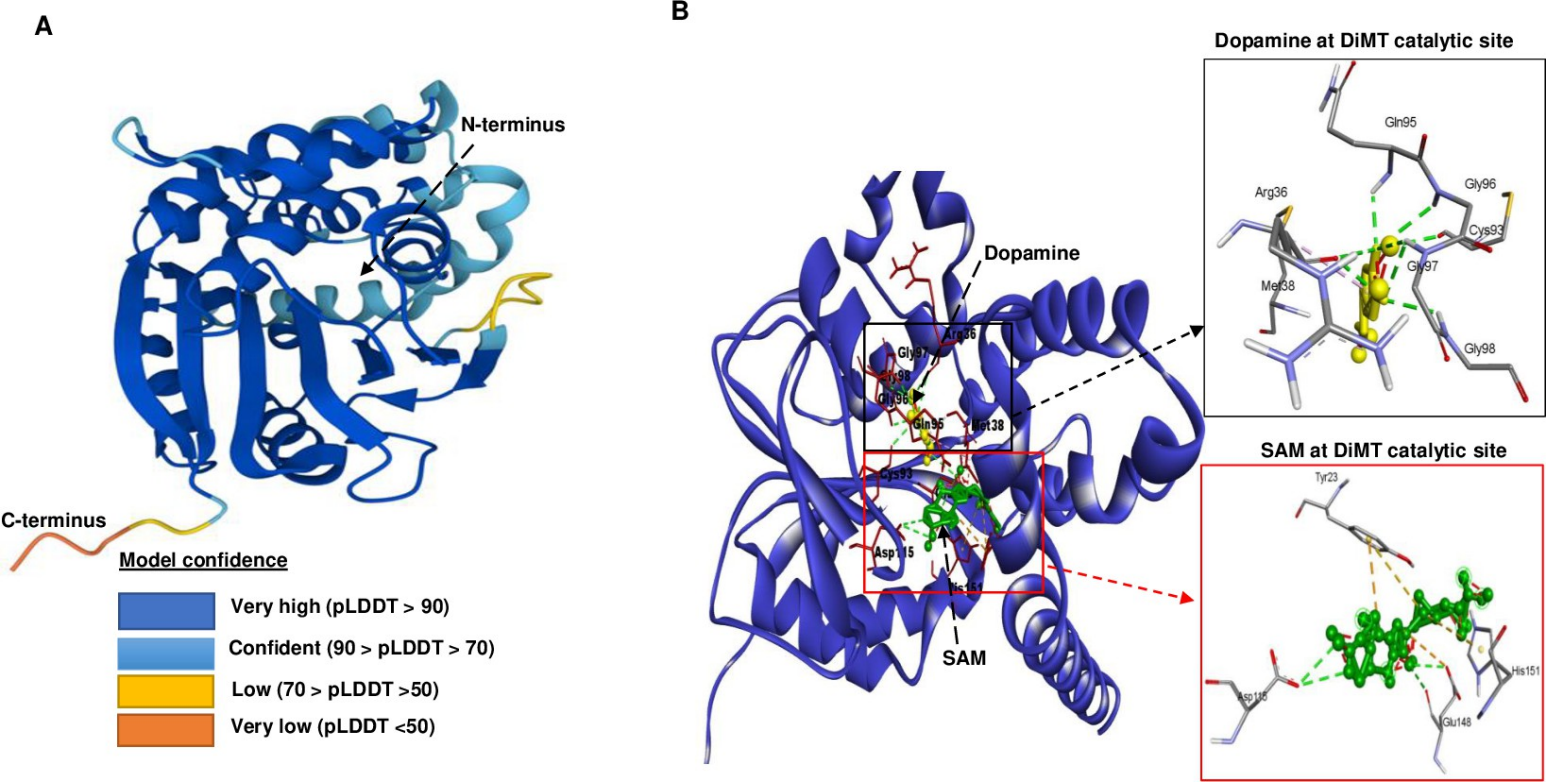
Predicted 3-dimensional structure of DiMT protein (UniParc ID. nDi.2.2.2.t06229) and its substrate binding site. **(A)** The AlphaFold-2 predicted DiMT protein structure with model confidence elaborated by the local distance difference test (pLDDT scores) and representative color codes. Dark blue: very high confidence with pLDDT > 90; sky blue: confident with 90 > pLDDT > 70; yellow: low confidence with 70 > pLDDT > 50; red: very low confidence with pLDDT < 50). **(B)** Docking prediction of the binding of co-substrate *S*-adenosyl-L-methionine (SAM) and substrate (dopamine) to amino acid residues within the DiMT methyltransferase domain, respectively. Docking results were visualized using Discovery Studio visualizer (BIOVIA Discovery Studio 2020 Client). Enlarged views of docked SAM and dopamine at the DiMT catalytic model are depicted on the right. Green dashed lines indicate hydrogen bonds with amino acid residues. The central thick stick green color structure indicates SAM whereas the stick yellow color indicates the dopamine substrate, respectively.

To predict the 3D-structure of DiMT protein, we performed *in silico* modelling employing local distance difference test (pLDDT) scores [20] and color codes for residues to delineate the model’s AF2-predicted configuration [19] (Fig 2A). In the AF2 model, residues with pLDDT > 90 indicated exceptionally high model confidence, whereas residues with pLDDT < 50 showed very low confidence. For the DiMT protein model, the majority of structural residues had pLDDT > 90. The model accuracy was further verified using a Ramachandran plot generated with PROCHECK system [21] which showed that out of 274 amino acid residues, 230 residues (92.7%) were in the most favored region, while 15 residues (6.0%) were in the allowed region (S1 Fig). With over 90% of amino acid residues being placed in the most favored region, our findings suggest that the generated model was accurate and reliable. By utilizing the PDBsum protein analysis system [21], we found that the secondary structure of DiMT protein possesses seven β-strands, with six of them being arranged in parallel, while one was coupled to the C-terminal in the antiparallel direction (S2 Fig). Importantly, the β-strands of DiMT were ordered in a 3214576 format, consistent with that displayed by the validated human COMT (PDB code: 3BWM) (S2 Fig). By COACH system analysis [22], the 3D-structure of DiMT possessed an *S*-adenosyl-L-methionine (SAM)-dependent methyltransferase catalytic domain, with residues 58–238 being the ligand binding amino acids (Fig 2B). Further, by blind docking, SAM and dopamine (a catecholamine substrate) were found to dock at distinct sites in close proximity to each other within the catalytic domain (Fig 2B) with high binding affinities (kcal/mol) of -9.3, and -6.9, respectively (Table 1). SAM formed hydrogen bonds with Asp-115 and Glu-148 amino acid residues, whereas dopamine formed hydrogen bonds with Arg-36, Met-38, Cys-93, Gln-95, Gly-96, Gly-97, Gly-98 amino acid residues. These findings supported the presence of two separate active sites for substrate and co-substrate, respectively, within the SAM-dependent methyltransferase catalytic domain.

**Table 1:** Docking parameters for substrate, co-substrate and inhibitors on DiMT catalytic domain.

Ligand	Binding energy (kcal/mol)	Residues interacting with ligand	Residues forming hydrogen bonds
SAM (co-substrate)	-9.3	Tyr-23, Asp-115, Glu-148, His-151	Asp-115, Glu-148
Dopamine (substrate)	-6.9	Arg-36, Met-38, Cys-93, Gln-95, Gly-96, Gly-97, Gly-98	Arg-36, Met-38, Cys-93, Gln-95, Gly-96, Gly-97, Gly-98
NSC56410 (inhibitor)	-9.9	Met-38, Gly-92, Asp-115, Ile-147, Glu-148	Met-38, Gly-92, Ile-147, Glu-148
NSC177383 (inhibitor)	-14.3	His-151, Arg-179, Lys-257	Lys-257
NSC145612 (inhibitor)	-14.5	His-151, Gln-183, Lys-257	Lys-257

### 3.3. Recombinant DiMT protein possesses COMT catalytic activity

**Fig 3:**
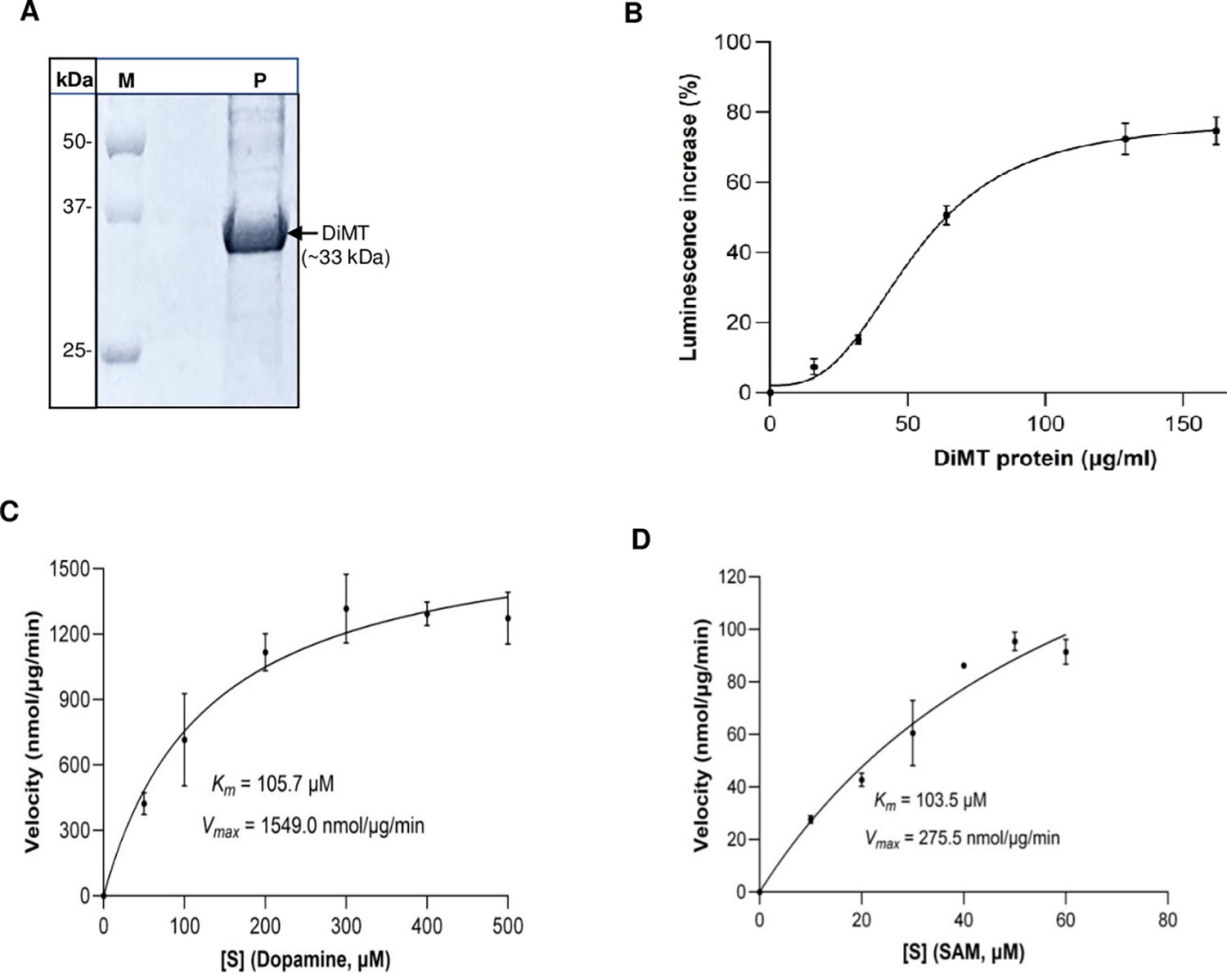
Analysis of purified recombinant DiMT protein and its enzyme kinetics. **(A)** SDS-PAGE analysis of the nickel affinity column chromatography-purified DiMT protein stained with coomassie blue (Lane M: protein ladder; Lane P: DiMT protein shown as a band of ~33 kDa). **(B)** Titration of purified recombinant DiMT protein in the MTase-Glo methyltransferase assay for the methylation of dopamine (360 μM) with SAM (30 μM) as methyl donor. **(C and D)** Enzyme kinetics of DiMT protein on substrate (dopamine) and co-substrate (SAM), respectively. The data shown represent the mean of three independent experiments with standard error bars.

To determine the *in vitro* enzymatic activity and kinetic parameters of DiMT, we expressed the His-tagged (C-terminus) recombinant DiMT protein in the PichiaPink eukaryotic expression system and purified it by affinity column chromatography in its native form. Analysis of the purified recombinant protein by SDS-PAGE showed that it was abundantly expressed and gel-fractionated into a band of approximately 33 kDa (Fig 3A), consistent with its expected molecular weight. To determine the enzymatic activity of the recombinant DiMT protein, we customized the commercial MTase-Glo Methyltransferase Activity Assay kit (Promega) in which we used recombinant DiMT protein as enzyme, SAM as the methyl donor and a catecholamine (dopamine) as substrate. We found that DiMT catalyzed the transfer of a methyl group from SAM to dopamine in a protein concentration-dependent manner, with 90 μg/ml of DiMT in the reaction being optimal (Fig 3B). Subsequently, kinetic parameters of DiMT on SAM and dopamine were determined by varying the SAM concentration, while maintaining a fixed concentration of dopamine and vice versa. DiMT showed catalytic activities following the Michaelis-Menten kinetics on the substrate dopamine (Fig 3C) and on the co-substrate SAM (Fig 3D). The obtained enzyme kinetic parameters of DiMT are summarized in Table 2. Raw data obtained from the DiMT’s concentration-dependent activity, and the Michaelis-Menten kinetics assays are presented in S1–S3 Tables.

**Table 2:** DiMT enzyme kinetics on substrate and co-substrate.

Substrate	*K*_*m*_ (μM)	*V*_*max*_ (nmol/μg/min)	*K*_*cat*_ (S^-1^)	*K*_*cat*_/ *K*_*m*_ (S^-1^/M^-1^)
SAM	103.5 ± 2.7	275.7 ± 3.9	42.94	4.1 × 10^5^
Dopamine	105.7 ± 6.2	1549.0 ± 21.4	241.28	2.8 × 10^6^

**Fig 4:**
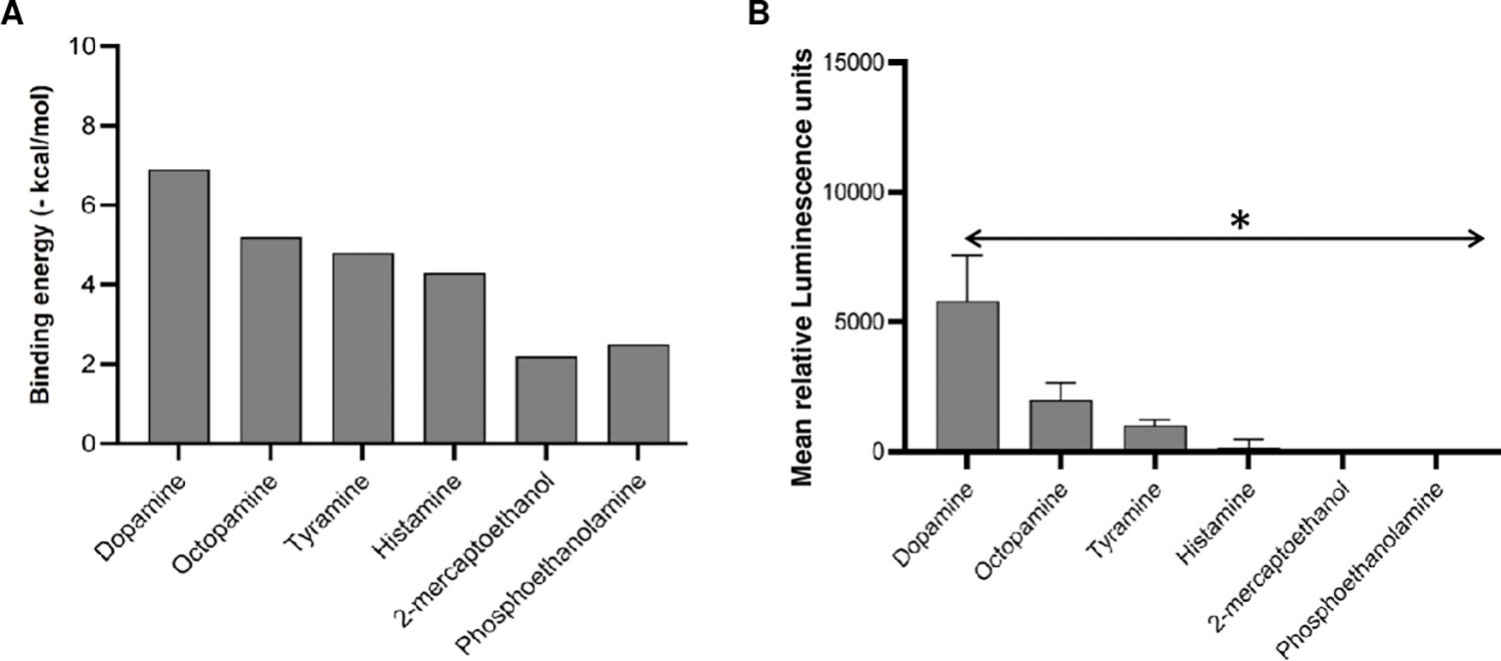
Analysis of the substrate specificity of DiMT protein catalytic activity. **(A)** Comparative *in silico* binding affinities (-kcal/mol) of various methyltransferase substrates at the catalytic site of DiMT protein. **(B)** *In vitro e*nzymatic activity of DiMT protein using various types of methyltransferase substrates with luminescence as a readout. Activity of DiMT with dopamine as substrate was significantly higher (*P < 0.0001) than those with other non-catecholamine substrates. The data shown is representative of the means from three independent experiments with standard error bars.

To determine the substrate specificity of DiMT’s catalytic activity, we initially performed molecular docking using DiMT as a macromolecule and different types of methyltransferase substrates as ligands. Dopamine showed higher binding affinity to the DiMT catalytic domain than non-catecholamine substrates (Octopamine, tyramine, 2-mercaptoethanol, phosphoethanolamine and histamine) (Fig 4A and S4 Table). Corroboratively, *in vitro* COMT enzymatic assays using DiMT as enzyme and SAM as co-substrate showed that, while DiMT readily catalyzed the methylation of dopamine, it did not depict catalytic activity for the methylation of non-catecholamine substrates (histamine, 2-mercaptolethanol and phosphoethanolamine) that are more structurally distant from catecholamines, but did show modest activity with octopamine and tyramine that are structurally very similar to catecholamines (Fig 4B and S5 Table). Collectively, these findings suggest that DiMT has substrate specificity for catecholamines.

### 3.4. Chemical inhibitors for DiMT enzymatic activity

We have previously reported a set of seven broad-spectrum chemical inhibitors for nematode phosphoethanolamine methyltransferases (PMTs) [30]. PMTs function by catalyzing the transfer of a methyl group from SAM (co-substrate) to phosphoethanolamine (substrate), which is similar to the COMT catalytic activity except for the difference in the substrate. Noteworthy, while PMTs are conserved in round worms, there are no orthologs of PMTs in parasitic filarial nematodes. Therefore, we tested 7 PMTs inhibitors (their chemical structures are depicted in S3 Fig) against the activity of DiMT and found that they depicted concentration-dependent inhibition which facilitated the derivation of their inhibitory IC_50_ values against the enzymatic activity of DiMT (Table 3). The raw data used to generate the inhibitory IC_50_ values of the inhibitors against DiMT are presented in S24–S30 Tables

**Table 3:** IC_50_ values for the inhibitors of DiMT catalytic activity.

Inhibitor	NSC35676	NSC62709	NSC177383	NSC145612	NSC133100	NSC56410	NSC87511
**IC**_**50**_ **(μM ± SEM)**	25.2 ± 1.4	76.2 ± 1.7	140.1 ± 2.2	62.1 ± 1.8	496.9 ± 2.7	300.0 ± 2.5	155.2 ± 2.2

### 3.5. DiMT inhibitors with efficacy against *D*. *immitis* microfilariae

**Fig 5:**
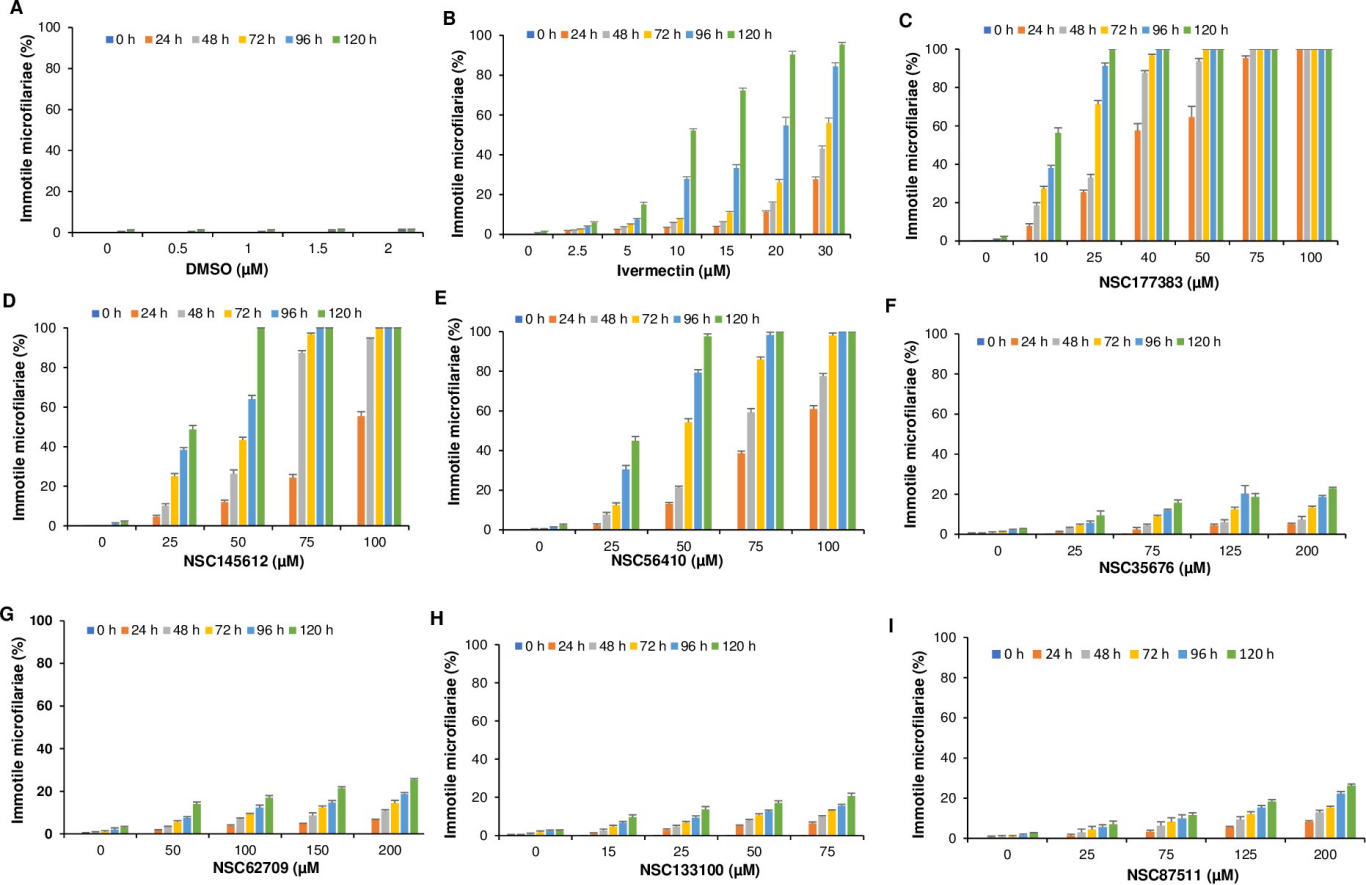
*In vitro* culture analysis of the effect on live *D*. *immitis* microfilariae of varying concentrations of (**A**) Dimethyl sulfoxide (DMSO), (**B**) Ivermectin (**C**) NSC177383, **(D)** NSC145612, (**E**) NSC56410, (**F**) NSC35676, (**G**) NSC62709, (**H**) NSC133100, and (**I**) NSC87511. At different time points of culture (24, 28, 72, 96, and 120 h post-treatment), 100 microfilariae in each culture were randomly counted, and the percentage of completely immotile microfilariae normalized to the cultures treated with corresponding volumes of DMSO was determined. The data shown represent means of three independent experiments with standard error bars.

The 7 DiMT inhibitors (Table 3) were next tested for *in vitro* efficacy against *D*. *immitis* microfilariae freshly extracted from dog peripheral blood. In our previous work, we had derived the PMT inhibitors’ cytotoxicity IC_50_ values in mammalian cells [30]. Therefore, for each compound, the highest concentration tested against microfilariae was at least 50% less than the respective compound’s cytotoxicity IC_50_. Initially, the effect of varying DMSO volumes (solvent used for reconstituting the compounds) on microfilariae were tested. We found that treatment of microfilariae with varying concentrations of DMSO in culture, up to a maximum used in compound-treated cultures (2% v/v) had no effect on the motility, activity, and survival of microfilariae for 120 h in culture (Fig 5A). This indicated that DMSO was non-toxic to microfilariae at the concentrations it was used as a solvent for the test compounds. Analysis of the effect of varying concentrations of ivermectin (the positive control drug) depicted both a dose-dependent and time-dependent effect on the inactivation of microfilariae, with 100% dead microfilariae being attained after 120 h of culture with 30 μM of ivermectin (Fig 5B). However, it is noteworthy that the concentrations of ivermectin we used for these *in vitro* studies are much higher than those that have been reported to be effective *in vivo* since the host animal’s immune system most likely contributes to the inactivation of the microfilariae post-treatment. Among the 7 compounds tested, 3 (NSC177383, NSC155612, and NSC56410) were found to have killing effect on microfilariae. NSC177383 showed the most robust concentration-dependent and time-dependent effect at relatively lower concentrations, with 25 μM killing 100% of the microfilariae after 120 h of culture (Fig 5C). Compound NSC145612 also had both concentration-dependent and time-dependent effect, with 50 μM killing 100% of the microfilariae after 120 h of culture (Fig 5D). Compound NSC56410 was found to have a concentration-dependent and time-dependent effect, with 50 μM killing 100% of the microfilariae after 120 h of culture (Fig 5E). The rest of the compounds (NSC35676, NSC62709, NSC133100 and NSC87511) had insignificant effect on microfilariae (Fig 5F–5I). All the raw data obtained from the *in vitro* anti-microfilariae assays and used to generate Fig 5A–5I are presented in S6–S23 Tables, respectively.

**Fig 6:**
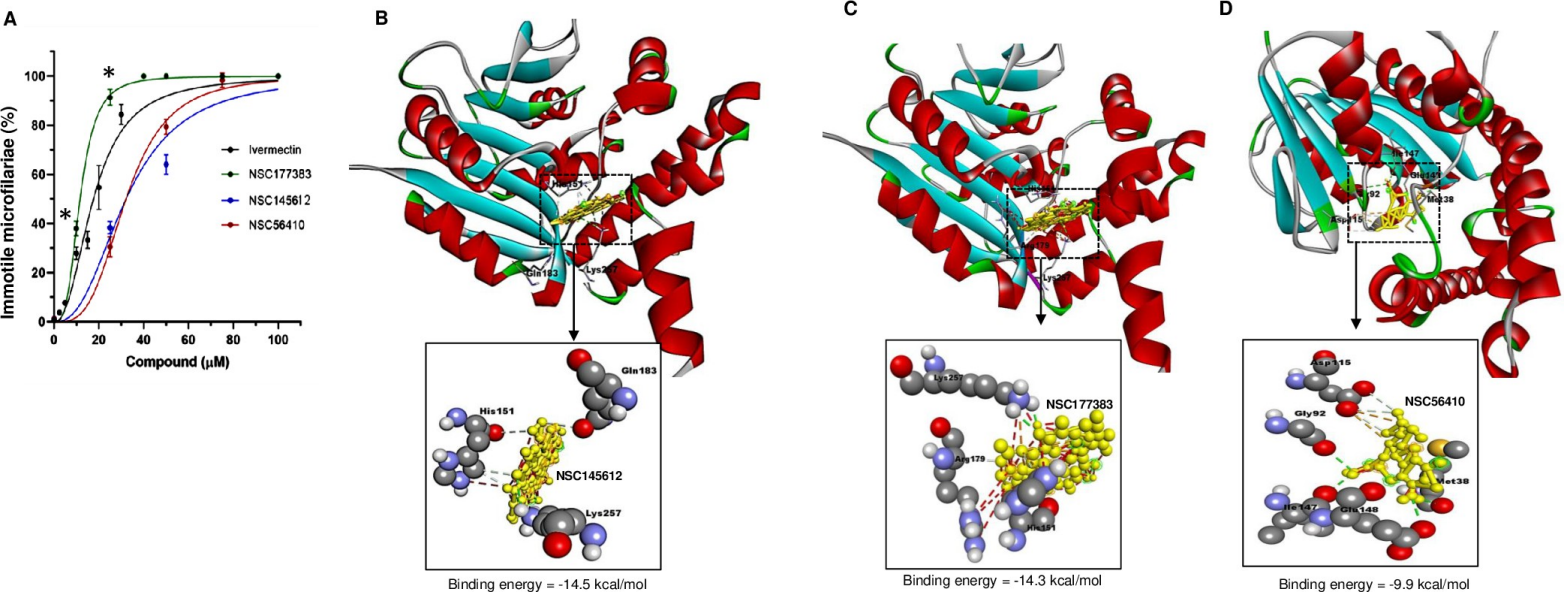
Analysis of the anti-parasite effect of DiMT inhibitors and their molecular interactions with the enzyme. (**A**) Comparison of the effect of varying concentrations of ivermectin (black), NSC177383 (green), NSC145612 (blue) and NSC56410 (red) on motility of *D*. *immitis* microfilariae after 96 h of culture. For each culture, 100 microfilariae were randomly counted, and the percentages of completely immotile microfilariae normalized to cultures treated with corresponding volumes of DMSO were determined. The data shown represent means of three independent experiments with standard error bars. NSC177383 had significantly higher efficacy (*, *P* < 0.05) than ivermectin at concentrations between 10 and 40 μM. The data shown represent the means from triplicate assays with standard error bars. (**B-D**) Docking prediction of the binding of (**B**) NSC145612, (**C**) NSC177383, and (**D**) NSC56410 to amino acid residues within the DiMT methyltransferase catalytic domain. Close-up views of the compounds at the DiMT catalytic model with the estimated binding energy are depicted at the bottom of each model. Structure of bound inhibitor is depicted by yellow stick structure with its interacting hydrogen bonds elaborated with green dashes. Red circle: hydrogen atom; Gray circle: carbon atom; Green circle: nitrogen atom).

Because the compound with the highest efficacy (NSC177383) did not show a significant difference in the percentage of dead microfilariae between 96 h and 120 h time-points (Fig 5C), data at 96 h post-treatment time point for all compounds was compared in a single plot. The results indicated that compound NSC177383 was more effective than the positive control drug (ivermectin) in inactivating microfilariae at lower concentrations (Fig 6A). On the other hand, compounds NSC145612 and NSC56410 were also effective, though at higher concentrations than that of ivermectin (Fig 6A). Data from this time-point was also used to derive the compounds’ antiparasitic EC_50_ concentrations using nonlinear regression analysis by GraphPad Prism software version 9.0 program. NSC177383 had a lower anti-parasite EC_50_ (11.65 μM) than ivermectin (17.61 μM), indicating that it was more efficacious than ivermectin against *D*. *immitis* microfilariae *in vitro* (Table 4). On the other hand, NSC145612 and NSC56410 had anti-parasite EC_50_ values of 32.16 and 32.15 μM, respectively (Table 4). Based on the cytotoxicity IC_50_ concentrations of NSC177383, NSC145612, NSC56410, and ivermectin in mammalian cells, we derived their Selectivity Indexes (SI). We found that the three test compounds (NSC177383, NSC145612, and NSC56410) all had SI values that were greater than 1 and several-fold larger than that of ivermectin (Table 4). This indicated that NSC177383, NSC145612, and NSC56410 are nontoxic to mammalian cells at their effective concentrations and are significantly more tolerable than ivermectin. To determine whether the non-motile microfilariae in the treated cultures were dead, after observation at 120 h post-treatment, the test compounds were washed out of the cultures using medium without compounds and the worms cultured for a further 72 h while observing them every 12 h to determine if they showed any sign of motility. Throughout the observation period, the microfilariae remained completely immotile and showed signs of disintegrating, indicating that they were dead, and that the inhibitors had a killing or parasiticidal effect against the microfilariae.

**Table 4:** Cytotoxicity IC_50_, anti-parasitic EC_50_ and Selectivity index values for test compounds.

Compound	Cytotoxicity IC_50_ (μM)	Anti-parasitic EC_50_ (μM)	Selectivity Index (SI)
NSC177383	85.5^a^	11.6	7.4
NSC145612	302.4^a^	32.2	9.4
NSC56410	188.4^a^	32.2	5.9
Ivermectin	37.4^b^	17.6	2.1

^a^Reported by Zhang et al., [30]; ^b^Reported by Tan et al., [31]

### 3.6. Chemical inhibitors with anti-parasite efficacy bind at DiMT catalytic site with high affinity

To determine the molecular interactions between the inhibitors and the DiMT protein’s catalytic domain, we performed molecular docking of the inhibitors with efficacy against *D*. *immitis* microfilariae. The ligand-receptor interactions were assessed using binding energy score, and hydrogen bond formation. The lower the binding affinity value, the stronger the binding interactions. Compounds NSC177383, NSC145612, and NSC56410 were found to bind favorably at the DiMT catalytic domain (Fig 6B and 6C). NSC145612 bound at the DiMT catalytic domain with the highest binding energy of -14.5 kcal/mol and hydrogen-bonded with residue lys-257 (Fig 6B). NSC177383 had the second highest binding energy of -14.3 kcal/mol and hydrogen-bonded with residues Asp-177 and Ile-147 (Fig 6C). NSC56410 had the least binding energy of—9.9kcal/mol, and hydrogen-bonded with DiMT residues Ile-147, Glu-148, Met-38, Gly-92, and Asp-115 (Fig 6D). Other docking interaction parameters for the compounds at the catalytic domain of DiMT including pi-cation/anion/alkyl binding are shown in Table 1.

## 4. Discussion

*D*. *immitis* is a mosquito-borne filarial nematode that causes a serious and potentially fatal disease in dogs called dirofilariasis. The low efficacy of existing treatments, combined with the growing threat of drug-resistant filarial nematodes, necessitate the urgent development of new anti-filarial treatments [32,33]. Catecholamines including dopamine and serotonin, are expressed in nematodes and have been shown to function as neurotransmitters or neuromodulators. However, excess amounts of dopamine and serotonin have been shown to inhibit nematode worm locomotion, pharyngeal pumping and fecundity [7–11,34]. Locomotion and pharyngeal pumping are essential activities for the survival of nematodes, while fecundity is critical to produce new progeny, [12]. Therefore, unregulated accumulation of catecholamines in nematodes would be deleterious to worm survival and reproduction. Catabolism of catecholamines in eukaryotic cells is achieved through methylation catalyzed by catechol-*O*-methyltransferases (COMT) [13–15]. Thus, the COMT-catalyzed methylation of catecholamines in nematodes would serve to abrogate the accumulation of active catecholamines and their deleterious effects on worms [34].

Herein, we describe the cloning, functional characterization and validation of a unique COMT (DiMT) as a viable drug molecular target in *D*. *immitis*. While COMT activity is also present in mammals, DiMT was selected based on presence of an ortholog in *C*. *elegans* that has an RNAi phenotype that is lethal, absence of a significant BLAST match (*E-*value ≤ 10^−5^) in the predicted proteomes of *Homo sapiens*, and predicted function as an enzyme [16]. Those parameters were corroborated by phylogenetic and homology analyses of DiMT that showed that it has conserved orthologs in other parasitic filarial nematodes including *B*. *malayi*, *L*. *loa*, *W*. *bancrofti* and *O*. *flexuous*, but is very distant from COMTs in mammalian species. Analysis of the DiMT amino acid sequence showed that it possesses a class-I SAM-dependent methyltransferase (SDMT) domain. The class-I SDMT domain fold has been shown to be highly conserved in COMTs [35], thus qualifying DiMT as a COMT. Importantly, DiMT showed significant disparities from mammalian COMTs, indicating that it can be a viable molecular drug target in parasitic filarial nematodes.

To gain insights into the molecular structure of DiMT and the configuration of its catalytic sites, we utilized structural bioinformatics tools and resources [36,37] to predict its 3D structure. The AF2 software that we used has capability to predict protein structures using primary amino acid sequences with appreciable accuracy [20,38,39]. Additionally, we employed a robust metric, the pLDDT [19], which depicted a very high confidence for the location of the amino acid residues within the DiMT structure. The accuracy of the model was corroborated using the Ramachandran plot statistics [40], which indicated that 92.7% of the residues in the model were located in the favored position. By comparison to the structure of human COMT that has been determined by crystallography, the secondary structure of DiMT depicted a structural fold like the one observed in human (PDB code: 3BWM) [41]. Further, DiMT exhibits a structural arrangement with seven beta-strands that is a conserved in COMTs [35]. To determine the specific molecular interactions between DiMT and ligands, we performed *in silico* molecular docking [42] of SAM and dopamine (as co-substrate and substrate, respectively) within the catalytic domain of DiMT. We found that dopamine bound to the glycine-rich G-loop (motif-I) and Asp or Glu-rich D-loop (motif-II) that are highly conserved within the SAM-dependent methyltransferase domain [43]. On the other hand, SAM bound to a distinct but proximal site to that of dopamine, indicating that DiMT protein possesses two functional active sites. Collectively, these predicted structural configurations and ligand interactions within DiMT’s catalytic domain strongly indicate that it is indeed a COMT.

To perform enzymatic functional characterization of DiMT, we used a eukaryotic expression system (*Pichia pastoris*) that facilitates post-translational protein processing and folding into native functional conformations [44,45]. Using an *in vitro* COMT assay [46] in which the natively purified recombinant DiMT protein served as enzyme, the methylation of the substrate (dopamine) was readily catalyzed in an enzyme concentration-dependent manner. Additionally, DiMT protein depicted Michaelis Menten kinetic parameters on the substrate and co-substrate, consistent with an active COMT [34,41]. Intriguingly, DiMT showed *K*_*m*_ value on dopamine that was several-fold lower than those reported for mammalian COMTs [47,48], suggesting that DiMT has different substrate affinities from those of its mammalian counterparts. Because eukaryotes express several structurally distinct families of SAM-dependent methyltransferases with varied substrates [2], we endeavored to compare DiMT’s activities on catecholamine and non-catecholamine substrates. While DiMT lacked activity of methylating non-catecholamine methyltransferase substrates (histamine, phosphoethanolamine, and 2-mercaptoethanol), it depicted very high activity for dopamine. Interestingly, DiMT possessed activity for octopamine and tyramine, but those activities were 3-fold and 6-fold lower than that of dopamine, respectively, which can be attributed to their close structural similarities with dopamine. Intriguingly, by using *in silico* molecular docking to compare the substrate binding energies at DiMT catalytic site, we found that dopamine had the highest binding affinity, followed by octopamine, tyramine, histamine, phosphoethanolamine and 2-mercaptoethanol, in that order, consistent with the observed *in vitro* enzymatic assay activities. Collectively, those observations underscored the specificity of DiMT as a COMT enzyme with a strict criterion that the substrate must possess a catechol structure [35]. However, it is noteworthy that these are *in vitro* studies using a recombinant DiMT protein, and that there may be variations in its configurations and activity from that expressed natively in the filarial nematodes.

Motility is the most common phenotype used in screening systems for anthelmintic activity [49,50], and potent commercially available anthelmintic drugs that induce paralysis of the body wall muscle of nematodes often exert a dose-response on motility [51–53]. Therefore, we used a motility assay to evaluate the DiMT inhibitors for efficacy against live *D*. *immitis*. Among the 7 inhibitors, we identified NSC177383, NSC145612 and NSC56410 that possessed efficacy against *D*. *immitis* microfilariae in a concentration-dependent manner. Among the three, NSC177383 showed an EC_50_ value for killing *D*. *immitis* that was about 1.5-fold lower than ivermectin and possessed a Selectivity Index (SI) that was 3.5-fold higher than that of ivermectin, indicating that it had better efficacy at lower tolerable concentrations in mammalian cells. On the other hand, while NSC145612 and NSC56410 both had EC_50_ values against *D*. *immitis* microfilariae that were 1.8-fold higher than ivermectin, they still possessed SI values that were 4.4-fold and 2.8-fold higher than ivermectin, respectively, suggesting that they were more tolerable than ivermectin in mammalian cells. Nevertheless, it is noteworthy that these selectivity indexes were obtained using *in vitro* systems, while under *in vivo* conditions the selectivity index for ivermectin is known to be more favorable.

By *in silico* molecular docking of NSC177383, NSC145612 and NSC56410 onto the DiMT protein model, NSC145612 and NSC177383 showed a similar binding affinity that was 1.5-fold stronger than that shown by NSC56410. This suggested that NSC145612 and NSC177383 were more stable in their bound configuration to DiMT which can translate into better inhibitory activity than that of NSC56410. Interestingly, NSC56410 was found to bind to some of the amino acid residues that bound to SAM at the catalytic site of DiMT, indicating that its mode of action is through competing with SAM for binding. On the other hand, while compounds NSC145612 and NSC177383 were found to bind within the catalytic domain of DiMT, they neither bound to SAM nor catecholamine substrate-specific amino acid residues, suggesting that their mode of inhibiting DiMT catalytic activity is non-competitive. Non-competitive inhibition patterns are common with bi-substrate enzymes [54–56] to which DiMT belongs (because it has a catecholamine as substrate and SAM as co-substrate). Importantly, non-competitive inhibitors tend to alter the enzyme’s active site conformation irrespective of its substrate *K*_*m*_ values, which in turn affects the enzyme’s activity [54,57]. Besides the binding affinity, hydrogen bond formation between receptor and ligand can contribute to the stability of the enzyme-ligand complex. All three compounds (NSC177383, NSC145612 and NSC56410) were found to form varying degrees of hydrogen bonds at the DiMT catalytic domain that could have enhanced the stability of the complex.

The inhibition of DiMT’s catalytic activity of catabolizing catecholamines through methylation would lead to excessive accumulation of active catecholamines in *D*. *immitis* microfilariae that would then disrupt cellular homeostasis, arrest pharyngeal pumping and locomotion, leading to death of the worms, as previously shown in the nematode *C*. *elegans* [7–11,34,58]. Importantly, NSC177383 and NSC145612 have been shown to possess drug-like properties. NSC177383, also known as tolypomycin-Y, is a natural antibiotic produced by *Streptomyces tolypophorus* that has strong antimicrobial activities against Gram-positive bacteria *in vitro* and *in vivo* and shows low toxicity in mice [59]. NSC145612 and NSC177383, are structurally similar to rifampin and its derivatives that are semisynthetic antibiotics with major activity against mycobacteria [29,30,60].

Collectively, our findings show that DiMT is an essential COMT that is conserved in parasitic filarial nematodes, but is significantly divergent from mammalian COMTs and, therefore, is a viable molecular drug target for the development of novel drugs against filarial nematode infections.

## Supporting information

S1 Table Titration of DiMT protein concentration in the MTase-Glo methyltransferase assay for the methylation of dopamine (360 μM) with SAM (30 μM) as methyl donor. (DOCX)

S2 Table Titration of SAM concentration in the MTase-Glo methyltransferase assay for the methylation of dopamine (360 μM) with DiMT as enzyme. (DOCX)

S3 Table Titration of dopamine concentration in the MTase-Glo methyltransferase assay with SAM (30 μM) as co-substrate and DiMT as enzyme. (DOCX)

S4 Table Comparative *in silico* binding affinities of various methyltransferase substrates at the catalytic site of DiMT protein. (DOCX)

S5 Table *In vitro* enzymatic activity of DiMT protein using various types of substrates. (DOCX)

S6 Table *In vitro* analysis of the effect of varying concentrations of DMSO on live *D*. *immitis* microfilariae. (DOCX)

S7 Table Mean values for the *in vitro* effect of varying concentrations of DMSO on live *D*. *immitis* microfilariae. (DOCX)

S8 Table *In vitro* analysis of the effect of varying concentrations of ivermectin on live *D*. *immitis* microfilariae. (DOCX)

S9 Table Mean values for the *in vitro* effect of varying concentrations of ivermectin on live *D*. *immitis* microfilariae. (DOCX)

S10 Table *In vitro* analysis of the effect of varying concentrations of NSC177383 on live *D*. *immitis* microfilariae. (DOCX)

S11 Table Mean values for the *in vitro* analysis of the effect of varying concentrations of NSC177383 on live *D*. *immitis* microfilariae. (DOCX)

S12 Table *In vitro* analysis of the effect of varying concentrations of NSC145612 on live *D*. *immitis* microfilariae. (DOCX)

S13 Table Mean values for the *in vitro* analysis of the effect of varying concentrations of NSC145612 on live *D*. *immitis* microfilariae. (DOCX)

S14 Table *In vitro* analysis of the effect of varying concentrations of NSC56410 on live *D*. *immitis* microfilariae. (DOCX)

S15 Table Mean values for the *in vitro* analysis of the effect of varying concentrations of NSC56410 on live *D*. *immitis* microfilariae. (DOCX)

S16 Table *In vitro* analysis of the effect of varying concentrations of NSC35676 on live *D*. *immitis* microfilariae. (DOCX)

S17 Table Mean values for the *in vitro* analysis of the effect of varying concentrations of NSC35676 on live *D*. *immitis* microfilariae. (DOCX)

S18 Table *In vitro* analysis of the effect of varying concentrations of NSC62709 on live *D*. *immitis* microfilariae. (DOCX)

S19 Table Mean values for the *in vitro* analysis of the effect of varying concentrations of NSC62709 on live *D*. *immitis* microfilariae. (DOCX)

S20 Table *In vitro* analysis of the effect of varying concentrations of NSC133100 on live *D*. *immitis* microfilariae. (DOCX)

S21 Table Mean values for the *in vitro* analysis of the effect of varying concentrations of NSC133100 on live *D*. *immitis* microfilariae. (DOCX)

S22 Table *In vitro* analysis of the effect of varying concentrations of NSC227186 on live *D*. *immitis* microfilariae. (DOCX)

S23 Table Mean values for the *in vitro* analysis of the effect of varying concentrations of NSC227186 on live *D*. *immitis* microfilariae. (DOCX)

S24 Table Inhibitory effect of varying concentrations of NSC35676 on the enzymatic activity of DiMT protein. (DOCX)

S25 Table Inhibitory effect of varying concentrations of NSC62709 on the enzymatic activity of DiMT protein. (DOCX)

S26 Table Inhibitory effect of varying concentrations of NSC177383 on the enzymatic activity of DiMT protein. (DOCX)

S27 Table Inhibitory effect of varying concentrations of NSC145612 on the enzymatic activity of DiMT protein. (DOCX)

S28 Table Inhibitory effect of varying concentrations of NSC133100 on the enzymatic activity of DiMT protein. (DOCX)

S29 Table Inhibitory effect of varying concentrations of NSC56410 on the enzymatic activity of DiMT protein. (DOCX)

S30 Table Inhibitory effect of varying concentrations of NSC227186 on the enzymatic activity of DiMT protein. (DOCX)

S1 Fig The Ramachandran plot of DiMT protein by PROCHECK server. (A) Plotting colors represent phi-psi backbone conformational areas: red represents the most favored regions, brown and yellow represent additional and generously allowed regions, while light-yellow patches represent regions that are not allowed. The sky-blue dots represent (φ, ψ) angles for each residue of the predicted structure. The α-helical (around 0, -45° and -75°, -45°) and β-sheet (near 135° and 180°) regions are highlighted with red color. Majority of the blue dots amino acid residues lie within the β-sheet and right-handed α-helix regions. (B) The Ramachandran plot statistics reveal that 92.7% of residues lie within the most favored region, indicating the reliability of the predicted model.
(PPTX)

S2 Fig Structural topology of the human COMT and DiMT proteins. (A) PDBsum-predicted secondary structural topology of human COMT (PDB code: 3BWM). 3-D structure of human COMT (right side) displaying the arrangement of β-strands (order 3214576) and the overall fold of the enzyme. (B) DiMT protein’s structural topology and 3-D structure displaying similar number of β-strands and arrangement of the overall fold of the human COMT protein structure. PyMoL (https://www.pymol.org) was used to visualize β-strands within the 3-D structure of COMT and DiMT.
(PPTX)

S3 Fig Illustrations of the chemical structures of the inhibitors for DiMT enzyme. (PPTX)

## References

[1] Schluckebier G, O’Gara M; Saenger W, Cheng XJ. Universal catalytic domain structure of AdoMet-dependent methyltransferases. Mol Biol. 1995; 247:16–20. doi: 10.1006/jmbi.1994.0117 7897657

[2] Schubert H L, Blumenthal RM, Cheng X. Many paths to methyltransfer: a chronicle of convergence. Trends Biochem Sci. 2003; 28:329–335. doi: 10.1016/S0968-0004(03)00090-2 12826405 PMC2758044

[3] Thakker DR, Creveling CR. O-methylation, p 191–230. In: Mulder GJ, editor. Conjugation Reactions in Drug Metabolism. London: Taylor & Francis, 1990.

[4] Weinshilboum R. Pharmacogenetics of methylation: relationship to drug metabolism. Clin Biochem. 1988; 21:201–210. doi: 10.1016/s0009-9120(88)80002-x 3044645

[5] Walsh CT, editor. Posttranscriptional Modification of Proteins: Expanding Nature’s Inventory. Grand Rapids (Michigan): Roberts & Co, 2006.

[6] Woodson LC, Weinshilboum RM. Human kidney thiopurine methyltransferase purification and biochemical properties. Biochem Pharmacol. 1983; 32:819–826. 6838629 10.1016/0006-2952(83)90582-8

[7] Ames MM, Selassie CD, Woodson LC, Van Loon JA, Hanch C, Weinshilboum RMJ. Thiopurine methyltransferase: structure-activity relationships for benzoic acid inhibitors and thiophenol substrates. J Med Chem. 1986; 29:354–358. doi: 10.1021/jm00153a009 3950915

[8] Schafer WR, Kenyon CJ. A calcium-channel homologue required for adaptation to dopamine and serotonin in *Caenorhabditis elegans*. Nature. 1995; 375:73–78. 7723846 10.1038/375073a0

[9] Rex E, Molitor SC, Hapiak V, Xiao H, Henderson M, Komuniecki R. Tyramine receptor (SER-2) isoforms are involved in the regulation of pharyngeal pumping and foraging behavior in *Caenorhabditis elegans*. J Neurochem. 2004; 91:1104–1115. 15569254 10.1111/j.1471-4159.2004.02787.x

[10] Rogers CM, Franks CJ, Walker RJ, Burke JF, Holden-Dye L. Regulation of the pharynx of *Caenorhabditis elegans* by 5-HT, octopamine, and FMRFamide-like neuropeptides. J Neurobiol. 2001; 49:235–244. 11745661 10.1002/neu.1078

[11] Niacaris T, Avery L. Serotonin regulates repolarization of the *C*. *elegans* pharyngeal muscle. J Exp Biol.2003; 206:223–231. 12477893 10.1242/jeb.00101 PMC4441752

[12] Chase DL, Koelle MR. *Biogenic amine neurotransmitters in C*. *elegans*. In: Community Research, editors. The *C*. *elegans*. Pasadena (CA): WormBook, 2007. 10.1895/wormbook.1.132.1 PMC4781333 18050501

[13] Veser J. Kinetics and inhibition studies of catechol O-methyltransferase from the yeast *Candida tropicalis*. J Bacteriol. 1987; 169:3696–3700. 3611026 10.1128/jb.169.8.3696-3700.1987 PMC212453

[14] Creveling CR, Thakker DR. 1994. O-, N-, and S-Methyltransferases. *In* Kauffman FC, Bock KW (ed), Conjugation-Deconjugation Reactions in Drug Metabolism and Toxicity: Handbook of Experimental Pharmacology, Springer-Verlag, New York.

[15] Vidgren J, Svensson LA, Lijas A. Nature. 1994; 368:354–358. 8127373 10.1038/368354a0

[16] Godel C, Kumar S, Koutsovoulos G, Ludin P, Nilsson D, Comandatore F, et al. The genome of the heartworm, *Dirofilaria immitis*, reveals drug and vaccine targets. FASEB J. 2012; 26: 4650–4661. 22889830 10.1096/fj.12-205096 PMC3475251

[17] Marchler-Bauer A, Lu S, Anderson JB, Chitsaz F, Derbyshire MK, DeWeese-Scott C, et al. CDD: A Conserved Domain Database for the functional annotation of proteins. Nucleic Acids Res. 2011; 39: D225–D229. doi: 10.1093/nar/gkq1189 21109532 PMC3013737

[18] Blum M, Chang HY, Chuguransky S, Grego T, Kandasaamy S, Mitchell A, et al. The InterPro protein families and domains database: 20 years on. Nucleic Acids Res. 2021; 49: D344–D354. doi: 10.1093/nar/gkaa977 33156333 PMC7778928

[19] Varadi M, Anyango S, Deshpande M, Nair S, Natassia C, Yordanova G, et al. AlphaFold Protein Structure Database: Massively expanding the structural coverage of protein-sequence space with high-accuracy models. Nucleic Acids Res. 2022; 50: D439–D444. doi: 10.1093/nar/gkab1061 34791371 PMC8728224

[20] Guo HB, Perminov A, Bekele S, Kedziora G, Farajollahi S, Varaljay V, et al. AlphaFold2 models indicate that protein sequence determines both structure and dynamics. Sci Rep. 2022; 12:10696. doi: 10.1038/s41598-022-14382-9 35739160 PMC9226352

[21] Laskowski RA, Jabłońska J, Pravda L, Vařeková RS, Thornton JM. PDBsum: Structural summaries of PDB entries. Protein Science. 2018; 27: 129–134. doi: 10.1002/pro.3289 28875543 PMC5734310

[22] Raj S, Sasidharan S, Dubey VK, Saudagar P. Identification of lead molecules against potential drug target protein MAPK4 from *L*. *Donovani*: An *in silico* approach using docking, molecular dynamics and binding free energy calculation. PLoS One. 2019; 14: e0221331. 31425543 10.1371/journal.pone.0221331 PMC6699710

[23] Zou L, Geng X, Li Z, Li T. Design of highly active substrates using molecular docking for microbial transglutaminase detection. RSC Adv. 2023; 13:5259–5265. doi: 10.1039/d2ra06467g 36793302 PMC9923216

[24] Hua L, Qianqian B, Jianfeng Z, Yinbiao X, Shengyu Y, Weishi X, et al. Directed evolution engineering to improve activity of glucose dehydrogenase by increasing pocket hydrophobicity. Front Microbiol. 2022; 13:1044226. doi: 10.3389/fmicb.2022.1044226 36439831 PMC9681798

[25] Shah-abadi ME, Ariaei A, Moradi F, Rustamzadeh A, Tanha RR, Sadigh N, et al. *In silico* Interactions of Natural and Synthetic Compounds with Key Proteins Involved in Alzheimer’s Disease: Prospects for Designing New Therapeutics Compound. Neurotox Res. 2023; 41: 408–430. 37086338 10.1007/s12640-023-00648-1 PMC10122091

[26] Kim S, Thiessen PA, Bolton EE, Chen J, Fu G, Gindulyte A, Han L, He J, He S, Shoemaker BA, Wang J, Yu B, Zhang J, Bryant SH. PubChem Substance and Compound databases. Nucleic Acids Res. 2016; 44: D1202–D13. doi: 10.1093/nar/gkv951 26400175 PMC4702940

[27] Wang YL, Suzek T, Zhang J, Wang J, He S, Cheng T, Shoemaker BA, Gindulyte A, Bryant SH. PubChem BioAssay: 2014 update. Nucleic Acids Res. 2014; 42: D1075–D82. doi: 10.1093/nar/gkt978 24198245 PMC3965008

[28] Daneial B, Joseph JPV, Ramakrishna G. Molecular dynamics simulation analysis of Focal Adhesive Kinase (FK) docked with solanesol as an anti-cancer agent. Bioinformation. 2017; 13: 274–283. 29081606 10.6026/97320630013274 PMC5651220

[29] Aliye M, Dekebo A, Tesso H, Abdo T, Eswaramoorthy R, Melaku Y. Molecular docking analysis and evaluation of the antibacterial and antioxidant activities of the constituents of *Ocimum cufodontii*. Sci Rep. 2021; 11:10101. 33980935 10.1038/s41598-021-89557-x PMC8115310

[30] Zhang X, Gianechini LS, Li K, Kaplan RM, Witola WH. Broad-Spectrum Inhibitors for Conserved Unique Phosphoethanolamine Methyltransferases in Parasitic Nematodes Possess Anthelmintic Efficacy. Antimicrob Agents Chemother. 2023; 67: e0000823. doi: 10.1128/aac.00008-23 37212658 PMC10269165

[31] Tan YL, Tan KSW, Chu JJH, Chow VT. Combination treatment with remdesivir and ivermectin exerts highly synergistic and potent antiviral activity against murine Coronavirus infection. Front Cell Infect Microbiol. 2021; 11:700502. doi: 10.3389/fcimb.2021.700502 34395311 PMC8362885

[32] Wolstenholme AJ, Evans CC, Jimenez PD, Moorhead AR. The emergence of macrocyclic lactone resistance in the canine heartworm, *Dirofilaria immitis*. Parasitology. 2015; 142: 1249–1259. 26040450 10.1017/S003118201500061X

[33] Bourguinat C, Lee ACY, Lizundia R, Blagburn BL, Liotta JL, Kraus MS, et al. Macrocyclic lactone resistance in *Dirofilaria immitis*: Failure of heartworm preventives and investigation of genetic markers for resistance. Vet Parasitol. 2015; 210:167–178. 25936435 10.1016/j.vetpar.2015.04.002

[34] Walker RJ, Holden-Dye L. Evolutionary aspects of transmitter molecules, their receptors and channels. Parasitology. 1991; 102: S7–S29. doi: 10.1017/s0031182000073261 1711668

[35] Ma Z, Liu H, Wu B. Structure-based drug design of catechol-O-methyltransferase inhibitors for CNS disorders. Br J Clin Pharmacol. 2014; 77:410–420. doi: 10.1111/bcp.12169 23713800 PMC3952716

[36] Bertoline MF, Lima AN, Krieger JE, Teixeira SK. Before and after AlphaFold2: An overview of protein structure prediction. Front Bioinform. 2023; 3:1120370. doi: 10.3389/fbinf.2023.1120370 36926275 PMC10011655

[37] Waman VP, Sen N, Varadi M, Daina A, Wodak SJ, Zoete V, et al. The impact of structural bioinformatics tools and resources on SARS-CoV-2 research and therapeutic strategies. Brief Bioinform. 2021; 22: 742–768. doi: 10.1093/bib/bbaa362 33348379 PMC7799268

[38] Tunyasuvunakool K, Adler J, Wu Z, Green T, Zielinski M, Žídek A, et al. Highly accurate protein structure prediction for the human proteome. Nature. 2021; 596: 590–596. doi: 10.1038/s41586-021-03828-1 34293799 PMC8387240

[39] Jumper J, Evans R, Pritzel A, Green T, Figurnov M, Ronneberger O, et al. Highly accurate protein structure prediction with AlphaFold. Nature. 2021; 96: 583–589. 10.1038/s41586-021-03819-2 PMC8371605 34265844

[40] Li D, Liu Y, Li K, Zhang L. Targeting F13 from monkeypox virus and variola virus by tecovirimat: Molecular simulation analysis. J Infect. 2022; 85: e99–e101. doi: 10.1016/j.jinf.2022.07.001 35810941

[41] Rutherford K, Le Trong I, Stenkamp RE, Parson WW. Crystal Structures of Human 108V and 108M Catechol O-Methyltransferase. J Mol Biol. 2008; 380:120–130. doi: 10.1016/j.jmb.2008.04.040 18486144

[42] Papanikolaou A, Chatzikonstantinou AV, Zarafeta D, Kourkoumelis N, Skretas G, Pavlidis Ι, et al. Substrate Specificity of the Highly Thermostable Esterase EstDZ3. ChemBioChem. 2023; 24: e202200642. doi: 10.1002/cbic.202200642 36545817

[43] Sun W, Xu X, Pavlova M, Edwards AM, Joachimiak A, Savchenko A. et al. The crystal structure of a novel SAM-dependent methyltransferase PH1915 from *Pyrococcus horikoshii*. Prot Sci. 2005; 14:3121–3128. 10.1110/ps.051821805 PMC2253237 16260766

[44] Wang H, Yang L, Liu M, Luo J. Protein post-translational modifications in the regulation of cancer hallmarks. Cancer Gene Ther. 2022; 30:526–547. doi: 10.1038/s41417-022-00464-3 35393571

[45] Wang X, Hong M. Protein Kinases and Cross-talk between Post-translational Modifications in the Regulation of Drug Transporters. Mol Pharmacol. 2023; 103:9–20. doi: 10.1124/molpharm.122.000604 36302660

[46] Yalcin D, Bayraktar O. Inhibition of catechol-O-methyltransferase (COMT) by some plant-derived alkaloids and phenolics. J Mol Catal B Enzym. 2010; 64:162–166.

[47] Su Y, DePasquale M, Liao G, Buchler I, Zhang G, Byers S, et al. Membrane-bound catechol-O-methyltransferase is the dominant isoform for dopamine metabolism in PC12 cells and rat brain. Eur J Pharmacol. 2021; 896: 173909. 33503461 10.1016/j.ejphar.2021.173909 PMC7962326

[48] Scott MC, Guerciolini R, Szumlanski C, Weinshilboum RM, Weinshilboum R. Mouse kidney histamine N-methyltransferase: Assay conditions, biochemical properties and strain variation. Agents Actions. 1991; 32:194–202. doi: 10.1007/BF01980873 1907425

[49] Mackenzie CD, Geary TG. Addressing the current challenges to finding new anthelminthic drugs. Expert Rev Anti Infect Ther. 2013; 11:539–541. doi: 10.1586/eri.13.49 23750723

[50] Partridge FA, Forman R, Bataille CJR, Wynne GM, Nick M, Russell AJ, et al. Anthelmintic drug discovery: target identification, screening methods and the role of open science. Beilstein J Org Chem. 2020; 16:1203–1224. doi: 10.3762/bjoc.16.105 32550933 PMC7277699

[51] Martin R. Modes of action of anthelmintic drugs. Vet J. 1997; 154:11–34. doi: 10.1016/s1090-0233(05)80005-x 9265850

[52] Várady M, Corba J. Comparison of six in vitro tests in determining benzimidazole and levamisole resistance in *Haemonchus contortus* and *Ostertagia circumcincta* of sheep. Vet Parasitol. 1999; 80:239–249. 9950347 10.1016/s0304-4017(98)00211-8

[53] George MM, Lopez-Soberal L, Storey BE, Howell SB, Kaplan RM. Motility in the L3 stage is a poor phenotype for detecting and measuring resistance to avermectin/milbemycin drugs in gastrointestinal nematodes of livestock. Int J Parasitol: Drugs Drug Resist 2018; 8:22–30. doi: 10.1016/j.ijpddr.2017.12.002 29274827 PMC6114081

[54] El Omari K, Bronckaers A, Liekens S, Pérez-Pérez MJ, Balzarini J, Stammers DK. Structural basis for non-competitive product inhibition in human thymidine phosphorylase: Implications for drug design. Biochem J. 2006; 399:199–204. doi: 10.1042/BJ20060513 16803458 PMC1609907

[55] Blat Y. Non-competitive inhibition by active site binders. Chem Biol Drug Des. 2010; 75:535–540. doi: 10.1111/j.1747-0285.2010.00972.x 20374252

[56] Freymann DM, Anne Wenck M, Engel JC, Feng J, Focia PJ, et al. Efficient identification of inhibitors targeting the closed active site conformation of the HPRT from *Trypanosoma cruzi*. Chem Biol. 2000; 7:957–968. 11137818 10.1016/s1074-5521(00)00045-4

[57] Knight ZA, Shokat KM. Features of selective kinase inhibitors. Chem Biol. 2005; 12:621–637. doi: 10.1016/j.chembiol.2005.04.011 15975507

[58] Horvitz HR, Chalfie M, Trent C, Sulston JE, Evans PD. Serotonin and octopamine in the nematode *Caenorhabditis elegans*. Science. 1979; 216:1012–1014. 10.1126/science.6805073 6805073

[59] Kishi T, Yamana H, Muroi M, Harada S, Asai M, Hasegawa T, et al. Tolypomycin, a new antibiotic. III: Isolation and characterization of tolypomycin Y. J. Antibiot. 1972; 25:11–15. 10.7164/antibiotics.25.11 4622052

[60] Floss HG, Yu TW. Rifamycin—Mode of action, resistance, and biosynthesis. Chem Rev. 2005; 105:621–632. doi: 10.1021/cr030112j 15700959

