# Supplementary material for: Characterization of a unique catechol-O-methyltransferase as a molecular drug target in parasitic filarial nematodes
Source: PLoS Negl Trop Dis. 2024 Aug 30;18(8):e0012473. doi: 10.1371/journal.pntd.0012473 (PMC11392244; doi:10.1371/journal.pntd.0012473)
Supplement: S1 Table — (DOCX) [file pntd.0012473.s001.docx]

**S1 Table**. Titration of DiMT protein concentration in the MTase-Glo methyltransferase assay for the methylation of dopamine (360 µM) with SAM (30 µM) as methyl donor

| **DiMT (µg/mL)** | **0** | **16** | **32** | **64** | **129** | **162** |
| --- | --- | --- | --- | --- | --- | --- |
| RLU^*^-1 | 0 | 3931.0 | 7905.0 | 18483.0 | 21800.0 | 26813.0 |
| RLU^*^-2 | 0 | 1850.0 | 5826.0 | 16262.0 | 25643.0 | 27283.0 |
| RLU^*^-3 | 0 | 3471.0 | 6266.0 | 17709.0 | 27077.0 | 24386.0 |
| **Mean RLU** | **0** | **3084.0** | **6665.7** | **17484.7** | **24840.0** | **26160.7** |
| **SEM** | **0** | **515.3** | **516.5** | **531.4** | **1286.3** | **732.9** |

*RLU, Relative Luminescence Units
