# Supplementary material for: Characterization of a unique catechol-O-methyltransferase as a molecular drug target in parasitic filarial nematodes
Source: PLoS Negl Trop Dis. 2024 Aug 30;18(8):e0012473. doi: 10.1371/journal.pntd.0012473 (PMC11392244; doi:10.1371/journal.pntd.0012473)
Supplement: S2 Table — (DOCX) [file pntd.0012473.s002.docx]

| **SAM (µM)** | **0** | **10** | **20** | **30** | **40** | **50** | **60** |
| --- | --- | --- | --- | --- | --- | --- | --- |
| RLU^*^-1 | 0 | 914.5 | 2199.5 | 2251.5 | 4459.5 | 4688.5 | 4881.5 |
| RLU^*^-2 | 0 | 723.5 | 2145.5 | 2688.5 | 4406.5 | 5346.5 | 5382.5 |
| RLU^*^-3 | 0 | 631.5 | 1782.5 | 4331.5 | 4327.5 | 5047.5 | 4183.5 |
| **Mean RLU** | 0 | **756.5** | **2042.5** | **3090.5** | **4397.8** | **5027.5** | **4815.8** |
| **SEM** | **0** | **68.1** | **106.9** | **517.0** | **31.3** | **155.3** | **283.9** |

**S2 Table**. Titration of SAM concentration in the MTase-Glo methyltransferase assay for the methylation of dopamine (360 µM) with DiMT as enzyme.

*RLU, Relative Luminescence Units
