# Supplementary material for: Characterization of a unique catechol-O-methyltransferase as a molecular drug target in parasitic filarial nematodes
Source: PLoS Negl Trop Dis. 2024 Aug 30;18(8):e0012473. doi: 10.1371/journal.pntd.0012473 (PMC11392244; doi:10.1371/journal.pntd.0012473)
Supplement: S3 Table — (DOCX) [file pntd.0012473.s003.docx]

| **Dopamine (uM)** | **0** | **50** | **100** | **200** | **300** | **400** | **500** |
| --- | --- | --- | --- | --- | --- | --- | --- |
| RLU^*^-1 | 0 | 106.0 | 322.0 | 958.0 | 1950.0 | 2877.0 | 2900.0 |
| RLU^*^-2 | 0 | 141.0 | 235.0 | 965.0 | 1857.0 | 2606.0 | 4023.0 |
| RLU^*^-3 | 0 | 96.0 | 617.0 | 1636.0 | 2677.0 | 3007.0 | 3526.0 |
| **Mean RLU** | **0** | **114.3** | **391.3** | **1186.3** | **2161.3** | **2830.0** | **3483.0** |
| **SEM** | **0** | **19.3** | **163.5** | **318.0** | **366.6** | **167.0** | **459.5** |

**S3 Table**. Titration of dopamine concentration in the MTase-Glo methyltransferase assay with SAM (30 µM) as co-substrate and DiMT as enzyme.

*RLU, Relative Luminescence Units
