# Supplementary material for: Characterization of a unique catechol-O-methyltransferase as a molecular drug target in parasitic filarial nematodes
Source: PLoS Negl Trop Dis. 2024 Aug 30;18(8):e0012473. doi: 10.1371/journal.pntd.0012473 (PMC11392244; doi:10.1371/journal.pntd.0012473)
Supplement: S4 Table — (DOCX) [file pntd.0012473.s004.docx]

**S4 Table.** Comparative *in silico* binding affinities of various methyltransferase substrates at the catalytic site of DiMT protein.

| **Substrate** | **Binding Energy (-kcal/mol)** |
| --- | --- |
| Dopamine | 6.9 |
| Octopamine | 5.2 |
| Tyramine | 4.8 |
| Histamine | 4.3 |
| 2-Mercaptoethanol | 2.2 |
| Phosphoethanolamine | 2.5 |
