# Supplementary material for: Characterization of a unique catechol-O-methyltransferase as a molecular drug target in parasitic filarial nematodes
Source: PLoS Negl Trop Dis. 2024 Aug 30;18(8):e0012473. doi: 10.1371/journal.pntd.0012473 (PMC11392244; doi:10.1371/journal.pntd.0012473)
Supplement: S5 Table — (DOCX) [file pntd.0012473.s005.docx]

**S5 Table.** *In vitro* enzymatic activity of DiMT protein using various types of substrates**.**

| **Substrate** | **Dopamine** | **Octopamine** | **Tyramine** | **Histamine** | **2-mercaptoethanol** | **Phosphoethanolamine** |
| --- | --- | --- | --- | --- | --- | --- |
| RLU^*^-1 | 9167.0 | 104.0 | 349.0 | 50.0 | -6143.0 | -3472.0 |
| RLU^*^-2 | 1832.0 | 279.0 | 1779.0 | 219.0 | -1595.0 | -3031.0 |
| RLU^*^-3 | 6420.0 | 5705.0 | 877.0 | 264.0 | -40.0 | 1152.0 |
| **Mean RLU** | **5806.3** | **1996.7** | **1001.7** | **167.3** | **-2592.7** | **-1783.7** |
| **SEM** | **1746.9** | **1501.2** | **340.9** | **53.2** | **1495.0** | **1203.0** |

*RLU, Relative Luminescence Units
