# Supplementary material for: Characterization of a unique catechol-O-methyltransferase as a molecular drug target in parasitic filarial nematodes
Source: PLoS Negl Trop Dis. 2024 Aug 30;18(8):e0012473. doi: 10.1371/journal.pntd.0012473 (PMC11392244; doi:10.1371/journal.pntd.0012473)
Supplement: S6 Table — (DOCX) [file pntd.0012473.s006.docx]

**S6 Table.** *In vitro* analysis of the effect of varying concentrations of DMSO on live *D. immitis* microfilariae.

| **DMSO** | **Completely Immotile Microfilariae (%)** | | | | | | | | | | | | | | | | | | | | | | |  |
| --- | --- | --- | --- | --- | --- | --- | --- | --- | --- | --- | --- | --- | --- | --- | --- | --- | --- | --- | --- | --- | --- | --- | --- | --- |
| **(% v/v)** | **0 h** | | | **24 h** | | | | **48 h** | | | | **72 h** | | | | **96 h** | | | | **120 h** | | | |  |
| 0.0 | 0 | 0 | 0 | | 0 | 0 | 0 | | 0 | 0 | 0 | | 0 | 0 | 0 | | 0 | 0 | 1 | | 1 | 0 | 2 | |
| 0.5 | 0 | 0 | 0 | | 0 | 0 | 0 | | 0 | 0 | 0 | | 0 | 0 | 0 | | 0 | 0 | 1 | | 1 | 0 | 2 | |
| 1.0 | 0 | 0 | 0 | | 0 | 0 | 0 | | 0 | 0 | 0 | | 0 | 0 | 0 | | 0 | 0 | 1 | | 1 | 0 | 2 | |
| 1.5 | 0 | 0 | 0 | | 0 | 0 | 0 | | 0 | 0 | 0 | | 0 | 0 | 0 | | 1 | 0 | 2 | | 1 | 1 | 2 | |
| 2.0 | 0 | 0 | 0 | | 0 | 0 | 0 | | 0 | 0 | 0 | | 0 | 0 | 0 | | 1 | 1 | 2 | | 1 | 1 | 2 | |
