# Supplementary material for: Characterization of a unique catechol-O-methyltransferase as a molecular drug target in parasitic filarial nematodes
Source: PLoS Negl Trop Dis. 2024 Aug 30;18(8):e0012473. doi: 10.1371/journal.pntd.0012473 (PMC11392244; doi:10.1371/journal.pntd.0012473)
Supplement: S7 Table — (DOCX) [file pntd.0012473.s007.docx]

**S7 Table.** Mean values for the *in vitro* effect of varying concentrations of DMSO on live *D. immitis* microfilariae.

| **DMSO** | **Mean completely immotile (%)** | | | | | | **SEM** | | | | | |
| --- | --- | --- | --- | --- | --- | --- | --- | --- | --- | --- | --- | --- |
| **(% v/v)** | **0 h** | **24 h** | **48 h** | **72 h** | **96 h** | **120 h** | **0 h** | **24 h** | **48 h** | **72 h** | **96 h** | **120 h** |
| 0.0 | 0 | 0 | 0 | 0 | 0.3 | 1.0 | 0 | 0 | 0 | 0 | 0.27 | 0.47 |
| 0.5 | 0 | 0 | 0 | 0 | 0.3 | 1.0 | 0 | 0 | 0 | 0 | 0.27 | 0.47 |
| 1.0 | 0 | 0 | 0 | 0 | 0.3 | 1.0 | 0 | 0 | 0 | 0 | 0.27 | 0.47 |
| 1.5 | 0 | 0 | 0 | 0 | 1.0 | 1.3 | 0 | 0 | 0 | 0 | 0.47 | 0.27 |
| 2.0 | 0 | 0 | 0 | 0 | 1.3 | 1.3 | 0 | 0 | 0 | 0 | 0.27 | 0.27 |
