# Supplementary material for: Characterization of a unique catechol-O-methyltransferase as a molecular drug target in parasitic filarial nematodes
Source: PLoS Negl Trop Dis. 2024 Aug 30;18(8):e0012473. doi: 10.1371/journal.pntd.0012473 (PMC11392244; doi:10.1371/journal.pntd.0012473)
Supplement: S8 Table — (DOCX) [file pntd.0012473.s008.docx]

| **Ivermectin** | **Completely Immotile Microfilariae (%)** | | | | | | | | | | | | | | | | | |
| --- | --- | --- | --- | --- | --- | --- | --- | --- | --- | --- | --- | --- | --- | --- | --- | --- | --- | --- |
| **(µM)** | **0 h** | | | **24 h** | | | **48 h** | | | **72 h** | | | **90 h** | | | **120 h** | | |
| 0 | 0 | 0 | 0 | 0 | 0 | 0 | 0 | 0 | 0 | 0 | 0 | 0 | 0 | 1 | 1 | 1 | 1 | 2 |
| 2.5 | 0 | 0 | 0 | 1 | 2 | 2 | 1.5 | 2.5 | 2 | 2 | 2.5 | 3 | 3 | 4 | 4.5 | 5 | 5.5 | 7 |
| 5.0 | 0 | 0 | 0 | 2 | 2 | 3 | 3 | 4 | 4 | 5 | 4.5 | 5.5 | 7.5 | 7 | 8.5 | 15 | 12.5 | 17.5 |
| 10.0 | 0 | 0 | 0 | 3 | 4 | 3 | 5 | 6 | 6 | 7.5 | 8.5 | 7.5 | 25 | 28.5 | 30 | 50 | 53.5 | 53 |
| 15.0 | 0 | 0 | 0 | 3.5 | 4.5 | 3.5 | 6 | 6.7 | 6 | 10 | 12 | 11 | 30 | 37 | 33 | 70 | 75 | 72 |
| 20.0 | 0 | 0 | 0 | 10 | 12.5 | 11 | 15 | 17 | 15 | 25 | 30 | 23 | 50 | 65 | 49 | 90 | 94 | 87 |
| 30.0 | 0 | 0 | 0 | 25 | 30 | 28 | 40 | 46 | 43 | 50 | 60 | 58 | 80 | 85 | 88 | 95 | 98 | 93 |

**S8 Table.** *In vitro* analysis of the effect of varying concentrations of ivermectin on live *D. immitis* microfilariae.
