# Supplementary material for: Characterization of a unique catechol-O-methyltransferase as a molecular drug target in parasitic filarial nematodes
Source: PLoS Negl Trop Dis. 2024 Aug 30;18(8):e0012473. doi: 10.1371/journal.pntd.0012473 (PMC11392244; doi:10.1371/journal.pntd.0012473)
Supplement: S9 Table — (DOCX) [file pntd.0012473.s009.docx]

**S9 Table.** Mean values for the *in vitro* effect of varying concentrations of ivermectin on live *D. immitis* microfilariae.

| **Ivermectin (µM)** | **Mean completely Immotile (%)** | | | | | | **SEM** | | | | | |
| --- | --- | --- | --- | --- | --- | --- | --- | --- | --- | --- | --- | --- |
|  | **0 h** | **24 h** | **48 h** | **72 h** | **96 h** | **120 h** | **0 h** | **24 h** | **48 h** | **72 h** | **96 h** | **120 h** |
| **0** | 0 | 0.0 | 0.0 | 0.0 | 0.7 | 1.33 | 0 | 0 | 0 | 0.0 | 0.3 | 0.3 |
| **2.5** | 0 | 1.7 | 2.0 | 2.5 | 3.8 | 5.83 | 0 | 0.3 | 0.2 | 0.3 | 0.4 | 0.5 |
| **5** | 0 | 2.3 | 3.7 | 5.0 | 7.7 | 15.0 | 0 | 0.3 | 0.3 | 0.3 | 0.4 | 1.2 |
| **10** | 0 | 3.3 | 5.7 | 7.8 | 27.8 | 52.2 | 0 | 0.3 | 0.3 | 0.3 | 1.2 | 0.9 |
| **15** | 0 | 3.8 | 6.2 | 11.0 | 33.3 | 72.3 | 0 | 0.3 | 0.2 | 0.5 | 1.7 | 1.2 |
| **20** | 0 | 11.2 | 15.7 | 26.0 | 54.7 | 90.3 | 0 | 0.6 | 0.5 | 1.7 | 4.2 | 1.7 |
| **30** | 0 | 27.7 | 43.0 | 56.0 | 84.3 | 95.3 | 0 | 1.2 | 1.4 | 2.5 | 1.9 | 1.2 |
