# Supplementary material for: Characterization of a unique catechol-O-methyltransferase as a molecular drug target in parasitic filarial nematodes
Source: PLoS Negl Trop Dis. 2024 Aug 30;18(8):e0012473. doi: 10.1371/journal.pntd.0012473 (PMC11392244; doi:10.1371/journal.pntd.0012473)
Supplement: S10 Table — (DOCX) [file pntd.0012473.s010.docx]

**S10 Table.** *In vitro* analysis of the effect of varying concentrations of NSC177383 on live *D. immitis* microfilariae.

| **NSC177383** | **Completely Immotile Microfilariae (%)** | | | | | | | | | | | | | | | | | |
| --- | --- | --- | --- | --- | --- | --- | --- | --- | --- | --- | --- | --- | --- | --- | --- | --- | --- | --- |
| **(µM)** | **0 h** | | | **24 h** | | | **48 h** | | | **72 h** | | | **96 h** | | | **120 h** | | |
| 0 | 0 | 0 | 0 | 0 | 0 | 0 | 0 | 0 | 0 | 0 | 0 | 0 | 0 | 1 | 1 | 1 | 2 | 3 |
| 10 | 0 | 0 | 0 | 5 | 8.5 | 10 | 15 | 19.5 | 21 | 25 | 27 | 30 | 35 | 38 | 41 | 50 | 61 | 58 |
| 25 | 0 | 0 | 0 | 25 | 28 | 23 | 30 | 37 | 32 | 70 | 76 | 68 | 90 | 95 | 89 | 100 | 100 | 100 |
| 40 | 0 | 0 | 0 | 50 | 65 | 58 | 85 | 90 | 88 | 95 | 98 | 97 | 100 | 100 | 100 | 100 | 100 | 100 |
| 50 | 0 | 0 | 0 | 60 | 78 | 56 | 90 | 95 | 96 | 100 | 100 | 100 | 100 | 100 | 100 | 100 | 100 | 100 |
| 75 | 0 | 0 | 0 | 95 | 93 | 98 | 100 | 100 | 100 | 100 | 100 | 100 | 100 | 100 | 100 | 100 | 100 | 100 |
| 100 | 0 | 0 | 0 | 100 | 100 | 100 | 100 | 100 | 100 | 100 | 100 | 100 | 100 | 100 | 100 | 100 | 100 | 100 |
