# Supplementary material for: Characterization of a unique catechol-O-methyltransferase as a molecular drug target in parasitic filarial nematodes
Source: PLoS Negl Trop Dis. 2024 Aug 30;18(8):e0012473. doi: 10.1371/journal.pntd.0012473 (PMC11392244; doi:10.1371/journal.pntd.0012473)
Supplement: S11 Table — (DOCX) [file pntd.0012473.s011.docx]

**S11 Table.** Mean values for the *in vitro* analysis of the effect of varying concentrations of NSC177383 on live *D. immitis* microfilariae.

| **NSC177383 (µM)** | **Mean completely Immotile (%)** | | | | | | **SEM** | | | | | |
| --- | --- | --- | --- | --- | --- | --- | --- | --- | --- | --- | --- | --- |
|  | **0 h** | **24 h** | **48 h** | **72 h** | **96 h** | **120 h** | **0 h** | **24 h** | **48 h** | **72 h** | **96 h** | **120 h** |
| **0** | 0 | 0 | 0 | 0 | 0.67 | 2 | 0 | 0 | 0 | 0 | 0.27 | 0.47 |
| **10** | 0 | 7.83 | 18.5 | 27.33 | 38 | 56.33 | 0 | 1.21 | 1.47 | 1.19 | 1.41 | 2.68 |
| **25** | 0 | 25.33 | 33 | 71.33 | 91.33 | 100 | 0 | 1.19 | 1.70 | 1.96 | 1.52 | 0 |
| **40** | 0 | 57.67 | 87.67 | 96.67 | 100 | 100 | 0 | 3.54 | 1.19 | 0.72 | 0 | 0 |
| **50** | 0 | 64.67 | 93.67 | 100 | 100 | 100 | 0 | 5.52 | 1.52 | 0 | 0 | 0 |
| **75** | 0 | 95.33 | 100 | 100 | 100 | 100 | 0 | 1.19 | 0 | 0 | 0 | 0 |
| **100** | 0 | 100 | 100 | 100 | 100 | 100 | 0 | 0 | 0 | 0 | 0 | 0 |
