# Supplementary material for: Characterization of a unique catechol-O-methyltransferase as a molecular drug target in parasitic filarial nematodes
Source: PLoS Negl Trop Dis. 2024 Aug 30;18(8):e0012473. doi: 10.1371/journal.pntd.0012473 (PMC11392244; doi:10.1371/journal.pntd.0012473)
Supplement: S12 Table — (DOCX) [file pntd.0012473.s012.docx]

**S12 Table.** *In vitro* analysis of the effect of varying concentrations of NSC145612 on live *D. immitis* microfilariae**.**

| **NSC145612** | **Completely Immotile Microfilariae (%)** | | | | | | | | | | | | | | | | | | |
| --- | --- | --- | --- | --- | --- | --- | --- | --- | --- | --- | --- | --- | --- | --- | --- | --- | --- | --- | --- |
| **(µM)** | **0 h** | | | **24 h** | | | **48 h** | | | **72 h** | | | **96 h** | | | | **120 h** | | |
| 0 | 0 | 0 | 0 | 0 | 0 | 0 | 0 | 0 | 0 | 0 | 0 | 0 | 2 | 1 | 0 | 3 | | 2 | 1 |
| 25 | 0 | 0 | 0 | 5 | 3 | 6 | 10 | 8 | 12.5 | 28 | 22 | 25 | 41 | 36 | 38 | 50 | | 44 | 52 |
| 50 | 0 | 0 | 0 | 10 | 12 | 14 | 25 | 23 | 31 | 40 | 44 | 46 | 60 | 64 | 68 | 100 | | 100 | 100 |
| 75 | 0 | 0 | 0 | 21 | 28 | 24 | 85 | 90 | 87 | 95 | 97 | 98 | 100 | 100 | 100 | 100 | | 100 | 100 |
| 100 | 0 | 0 | 0 | 50 | 56 | 60 | 93 | 95 | 95 | 100 | 100 | 100 | 100 | 100 | 100 | 100 | | 100 | 100 |
