# Supplementary material for: Characterization of a unique catechol-O-methyltransferase as a molecular drug target in parasitic filarial nematodes
Source: PLoS Negl Trop Dis. 2024 Aug 30;18(8):e0012473. doi: 10.1371/journal.pntd.0012473 (PMC11392244; doi:10.1371/journal.pntd.0012473)
Supplement: S13 Table — (DOCX) [file pntd.0012473.s013.docx]

**S13 Table.** Mean values for the *in vitro* analysis of the effect of varying concentrations of NSC145612 on live *D. immitis* microfilariae.

| **NSC145612 (µM)** | **Mean completely Immotile (%)** | | | | | | **SEM** | | | | | |
| --- | --- | --- | --- | --- | --- | --- | --- | --- | --- | --- | --- | --- |
|  | **0 h** | **24 h** | **48 h** | **72 h** | **96 h** | **120 h** | **0 h** | **24 h** | **48 h** | **72 h** | **96 h** | **120 h** |
| **0** | 0 | 0 | 0 | 0 | 1 | 2 | 0 | 0 | 0 | 0 | 0.47 | 0.47 |
| **25** | 0 | 4.67 | 10.17 | 25 | 38.33 | 48.67 | 0 | 0.72 | 1.06 | 1.41 | 1.19 | 1.96 |
| **50** | 0 | 12 | 26.33 | 43.33 | 64 | 100 | 0 | 0.94 | 1.96 | 1.44 | 1.89 | 0 |
| **75** | 0 | 24.33 | 87.33 | 96.67 | 100 | 100 | 0 | 1.66 | 1.19 | 0.72 | 0 | 0 |
| **100** | 0 | 55.33 | 94.33 | 100 | 100 | 100 | 0 | 2.37 | 0.54 | 0 | 0 | 0 |
