# Supplementary material for: Characterization of a unique catechol-O-methyltransferase as a molecular drug target in parasitic filarial nematodes
Source: PLoS Negl Trop Dis. 2024 Aug 30;18(8):e0012473. doi: 10.1371/journal.pntd.0012473 (PMC11392244; doi:10.1371/journal.pntd.0012473)
Supplement: S14 Table — (DOCX) [file pntd.0012473.s014.docx]

**S14 Table.** *In vitro* analysis of the effect of varying concentrations of NSC56410 on live *D. immitis* microfilariae**.**

| **NSC56410** | **Completely Immotile Microfilariae (%)** | | | | | | | | | | | | | | | | | |
| --- | --- | --- | --- | --- | --- | --- | --- | --- | --- | --- | --- | --- | --- | --- | --- | --- | --- | --- |
| **(µM)** | **0 h** | | | **24 h** | | | **48 h** | | | **72 h** | | | **96 h** | | | **120 h** | | |
| 0 | 0 | 0 | 0 | 0 | 0 | 0 | 0 | 1 | 0 | 0 | 1 | 0 | 1 | 2 | 1 | 3 | 3 | 1 |
| 25 | 0 | 0 | 0 | 2 | 4 | 1 | 8 | 10 | 5 | 15 | 12.5 | 10 | 35 | 29.5 | 27 | 50 | 44 | 41 |
| 50 | 0 | 0 | 0 | 15 | 11.5 | 12.5 | 23 | 21 | 20 | 56 | 50 | 57 | 80 | 76 | 82 | 100 | 95 | 98 |
| 75 | 0 | 0 | 0 | 36 | 40 | 40 | 55 | 60 | 63 | 83 | 88 | 87 | 100 | 100 | 95 | 100 | 100 | 100 |
| 100 | 0 | 0 | 0 | 60 | 58 | 65 | 75 | 78 | 80 | 95 | 100 | 99 | 100 | 100 | 100 | 100 | 100 | 100 |
