# Supplementary material for: Characterization of a unique catechol-O-methyltransferase as a molecular drug target in parasitic filarial nematodes
Source: PLoS Negl Trop Dis. 2024 Aug 30;18(8):e0012473. doi: 10.1371/journal.pntd.0012473 (PMC11392244; doi:10.1371/journal.pntd.0012473)
Supplement: S15 Table — (DOCX) [file pntd.0012473.s015.docx]

**S15 Table.** Mean values for the *in vitro* analysis of the effect of varying concentrations of NSC56410 on live *D. immitis* microfilariae.

| **NSC56410 (µM)** | **Mean completely Immotile (%)** | | | | | | **SEM** | | | | | |
| --- | --- | --- | --- | --- | --- | --- | --- | --- | --- | --- | --- | --- |
|  | **0 h** | **24 h** | **48 h** | **72 h** | **96 h** | **120 h** | **0 h** | **24 h** | **48 h** | **72 h** | **96 h** | **120 h** |
| **0** | 0 | 0 | 0.33 | 0.33 | 1.33 | 2.33 | 0 | 0 | 0.27 | 0.27 | 0.27 | 0.54 |
| **25** | 0 | 2.33 | 7.67 | 12.5 | 30.5 | 45 | 0 | 0.72 | 1.19 | 1.18 | 1.93 | 2.16 |
| **50** | 0 | 13 | 21.33 | 54.33 | 79.33 | 97.67 | 0 | 0.85 | 0.72 | 1.78 | 1.44 | 1.19 |
| **75** | 0 | 38.67 | 59.33 | 86 | 98.33 | 100 | 0 | 1.09 | 1.91 | 1.25 | 1.36 | 0 |
| **100** | 0 | 61 | 77.67 | 98 | 100 | 100 | 0 | 1.70 | 1.19 | 1.25 | 0 | 0 |
