# Supplementary material for: Characterization of a unique catechol-O-methyltransferase as a molecular drug target in parasitic filarial nematodes
Source: PLoS Negl Trop Dis. 2024 Aug 30;18(8):e0012473. doi: 10.1371/journal.pntd.0012473 (PMC11392244; doi:10.1371/journal.pntd.0012473)
Supplement: S17 Table — (DOCX) [file pntd.0012473.s017.docx]

**S17 Table.** Mean values for the *in vitro* analysis of the effect of varying concentrations of NSC35676 on live *D. immitis* microfilariae.

| **NSC35676 (µM)** | **Mean completely Immotile (%)** | | | | | | **SEM** | | | | | |
| --- | --- | --- | --- | --- | --- | --- | --- | --- | --- | --- | --- | --- |
|  | **0 h** | **24 h** | **48 h** | **72 h** | **96 h** | **120 h** | **0 h** | **24 h** | **48 h** | **72 h** | **96 h** | **120 h** |
| **0** | 0.33 | 0.33 | 0.67 | 1 | 2 | 2.67 | 0.27 | 0.27 | 0.54 | 0.47 | 0.47 | 0.27 |
| **25** | 0 | 1 | 3 | 4.33 | 5.67 | 9.33 | 0 | 0.47 | 0.47 | 0.72 | 0.98 | 2.33 |
| **75** | 0 | 2.33 | 4.33 | 9 | 12 | 15.67 | 0 | 1.09 | 0.72 | 0.47 | 0.47 | 1.44 |
| **125** | 0 | 4.33 | 6 | 12.33 | 20.33 | 18.67 | 0 | 0.72 | 1.25 | 1.19 | 3.95 | 1.66 |
| **200** | 0 | 5 | 7.33 | 13.33 | 18.67 | 23 | 0 | 0.47 | 1.52 | 0.72 | 0.72 | 0.47 |
