# Supplementary material for: Characterization of a unique catechol-O-methyltransferase as a molecular drug target in parasitic filarial nematodes
Source: PLoS Negl Trop Dis. 2024 Aug 30;18(8):e0012473. doi: 10.1371/journal.pntd.0012473 (PMC11392244; doi:10.1371/journal.pntd.0012473)
Supplement: S18 Table — (DOCX) [file pntd.0012473.s018.docx]

**S18 Table.** *In vitro* analysis of the effect of varying concentrations of NSC62709 on live *D. immitis* microfilariae**.**

| **NSC62709** | **Completely Immotile Microfilariae (%)** | | | | | | | | | | | | | | | | | |
| --- | --- | --- | --- | --- | --- | --- | --- | --- | --- | --- | --- | --- | --- | --- | --- | --- | --- | --- |
| **(µM)** | **0 h** | | | **24 h** | | | **48 h** | | | **72 h** | | | **96 h** | | | **120 h** | | |
| 0 | 0 | 0 | 0 | 0 | 0 | 1 | 0 | 1 | 1 | 0 | 1 | 2 | 1 | 1 | 4 | 3 | 2 | 4 |
| 50 | 0 | 0 | 0 | 1 | 2 | 2 | 3 | 4 | 3 | 5 | 7 | 5 | 7 | 9 | 6 | 12 | 14 | 16 |
| 100 | 0 | 0 | 0 | 3 | 3 | 5 | 7 | 6 | 8 | 9 | 9 | 10 | 10 | 12 | 15 | 15 | 17 | 19 |
| 150 | 0 | 0 | 0 | 5 | 4 | 5 | 9 | 6 | 11 | 12 | 11 | 14 | 13 | 14 | 17 | 20 | 21 | 23 |
| 200 | 0 | 0 | 0 | 7 | 6 | 7 | 12 | 9 | 11 | 16 | 11 | 16 | 19 | 17 | 20 | 24 | 26 | 26 |
