# Supplementary material for: Characterization of a unique catechol-O-methyltransferase as a molecular drug target in parasitic filarial nematodes
Source: PLoS Negl Trop Dis. 2024 Aug 30;18(8):e0012473. doi: 10.1371/journal.pntd.0012473 (PMC11392244; doi:10.1371/journal.pntd.0012473)
Supplement: S19 Table — (DOCX) [file pntd.0012473.s019.docx]

**S19 Table.** Mean values for the *in vitro* analysis of the effect of varying concentrations of NSC62709 on live *D. immitis* microfilariae.

| **NSC62709 (µM)** | **Mean completely Immotile (%)** | | | | | | **SEM** | | | | | |
| --- | --- | --- | --- | --- | --- | --- | --- | --- | --- | --- | --- | --- |
|  | **0 h** | **24 h** | **48 h** | **72 h** | **96 h** | **120 h** | **0 h** | **24 h** | **48 h** | **72 h** | **96 h** | **120 h** |
| 0 | 0 | 0.33 | 0.67 | 1 | 2 | 3 | 0 | 0.27 | 0.27 | 0.47 | 0.82 | 0.47 |
| 50 | 0 | 1.67 | 3.33 | 5.67 | 7.33 | 14 | 0 | 0.27 | 0.27 | 0.54 | 0.72 | 0.94 |
| 100 | 0 | 3.67 | 7 | 9.33 | 12.33 | 17 | 0 | 0.54 | 0.47 | 0.27 | 1.19 | 0.94 |
| 150 | 0 | 4.67 | 8.67 | 12.33 | 14.67 | 21.33 | 0 | 0.27 | 1.19 | 0.72 | 0.98 | 0.72 |
| 200 | 0 | 6.67 | 10.67 | 14.33 | 18.67 | 25.33 | 0 | 0.27 | 0.72 | 1.36 | 0.72 | 0.54 |
