# Supplementary material for: Characterization of a unique catechol-O-methyltransferase as a molecular drug target in parasitic filarial nematodes
Source: PLoS Negl Trop Dis. 2024 Aug 30;18(8):e0012473. doi: 10.1371/journal.pntd.0012473 (PMC11392244; doi:10.1371/journal.pntd.0012473)
Supplement: S21 Table — (DOCX) [file pntd.0012473.s021.docx]

**S21 Table.** Mean values for the *in vitro* analysis of the effect of varying concentrations of NSC133100 on live *D. immitis* microfilariae

| **NSC133100 (µM)** | **Mean completely Immotile (%)** | | | | | | **SEM** | | | | | |
| --- | --- | --- | --- | --- | --- | --- | --- | --- | --- | --- | --- | --- |
|  | **0 h** | **24 h** | **48 h** | **72 h** | **96 h** | **120 h** | **0 h** | **24 h** | **48 h** | **72 h** | **96 h** | **120 h** |
| **0** | 0.33 | 0.33 | 0.67 | 1.67 | 2.33 | 2.67 | 0.27 | 0.27 | 0.54 | 0.72 | 0.54 | 0.27 |
| **15** | 0 | 1 | 2.67 | 4.67 | 6.67 | 9.67 | 0 | 0.47 | 0.72 | 0.72 | 0.72 | 1.19 |
| **25** | 0 | 3 | 4.67 | 6.67 | 9.33 | 13.67 | 0 | 0.47 | 0.72 | 0.72 | 0.98 | 1.52 |
| **50** | 0 | 5 | 8 | 10.67 | 12.67 | 17 | 0 | 0.47 | 0.47 | 0.72 | 0.72 | 1.25 |
| **75** | 0 | 6.33 | 10 | 13 | 15.67 | 20.67 | 0 | 0.72 | 0.47 | 0.47 | 0.72 | 1.52 |
