# Supplementary material for: Characterization of a unique catechol-O-methyltransferase as a molecular drug target in parasitic filarial nematodes
Source: PLoS Negl Trop Dis. 2024 Aug 30;18(8):e0012473. doi: 10.1371/journal.pntd.0012473 (PMC11392244; doi:10.1371/journal.pntd.0012473)
Supplement: S23 Table — (DOCX) [file pntd.0012473.s023.docx]

**S23 Table.** Mean values for the *in vitro* analysis of the effect of varying concentrations of NSC227186 on live *D. immitis* microfilariae**.**

| **NSC227186 (µM)** | **Mean completely Immotile %** | | | | | | **SEM** | | | | | |
| --- | --- | --- | --- | --- | --- | --- | --- | --- | --- | --- | --- | --- |
|  | **0 h** | **24 h** | **48 h** | **72 h** | **96 h** | **120 h** | **0 h** | **24 h** | **48 h** | **72 h** | **96 h** | **120 h** |
| 0 | 0 | 0.67 | 1 | 1 | 1.67 | 2.67 | 0 | 0.54 | 0.47 | 0.47 | 0.54 | 0.27 |
| 25 | 0 | 1.33 | 3 | 4.33 | 5.67 | 7 | 0 | 0.72 | 1.70 | 1.66 | 1.19 | 1.70 |
| 75 | 0 | 3.33 | 6.33 | 8.33 | 10 | 11.67 | 0 | 0.72 | 1.96 | 1.96 | 1.70 | 1.09 |
| 125 | 0 | 5.67 | 9.33 | 12 | 15.33 | 18.33 | 0 | 0.27 | 1.52 | 1.25 | 0.98 | 0.98 |
| 200 | 0 | 8.33 | 13 | 15.33 | 22.33 | 26.33 | 0 | 0.54 | 0.94 | 0.72 | 0.98 | 0.72 |
