# Supplementary material for: Characterization of a unique catechol-O-methyltransferase as a molecular drug target in parasitic filarial nematodes
Source: PLoS Negl Trop Dis. 2024 Aug 30;18(8):e0012473. doi: 10.1371/journal.pntd.0012473 (PMC11392244; doi:10.1371/journal.pntd.0012473)
Supplement: S24 Table — (DOCX) [file pntd.0012473.s024.docx]

**S24 Table:** Inhibitory effect of varying concentrations of NSC35676 on the enzymatic activity of DiMT protein.

| **NSC35676 (µM)** | **0** | **15** | **25** | **50** | **75** | **100** |
| --- | --- | --- | --- | --- | --- | --- |
| **Mean Percent Inhibition** | 0 | 41.9 | 45.9 | 62.0 | 63.8 | 68.1 |
|  | 0 | 44.3 | 51.6 | 58.4 | 67.9 | 72.1 |
|  | 0 | 44.1 | 47.0 | 60.6 | 65.4 | 71.2 |
| **Average** | **0** | **43.4** | **48.2** | **60.4** | **65.7** | **70.5** |
| **SEM** | 0 | **0.6** | **1.4** | **0.9** | **1.0** | **1.0** |
