# Supplementary material for: Characterization of a unique catechol-O-methyltransferase as a molecular drug target in parasitic filarial nematodes
Source: PLoS Negl Trop Dis. 2024 Aug 30;18(8):e0012473. doi: 10.1371/journal.pntd.0012473 (PMC11392244; doi:10.1371/journal.pntd.0012473)
Supplement: S25 Table — (DOCX) [file pntd.0012473.s025.docx]

**S25 Table.** Inhibitory effect of varying concentrations of NSC62709 on the enzymatic activity of DiMT protein.

| **NSC62709 (µM)** | **0** | **40** | **80** | **120** | **140** | **180** |
| --- | --- | --- | --- | --- | --- | --- |
| **Mean Percent Inhibition** | 0 | 32.1 | 63.8 | 65.7 | 72.8 | 81.5 |
|  | 0 | 22.9 | 49.7 | 71.6 | 64.4 | 69.4 |
|  | 0 | 27.7 | 46.2 | 62.3 | 72.4 | 74.5 |
| **Average** | 0 | **27.6** | **53.2** | **66.5** | **69.9** | **75.1** |
| **SEM** | 0 | **2.2** | **4.4** | **2.2** | **2.2** | **2.9** |
