# Supplementary material for: Characterization of a unique catechol-O-methyltransferase as a molecular drug target in parasitic filarial nematodes
Source: PLoS Negl Trop Dis. 2024 Aug 30;18(8):e0012473. doi: 10.1371/journal.pntd.0012473 (PMC11392244; doi:10.1371/journal.pntd.0012473)
Supplement: S26 Table — (DOCX) [file pntd.0012473.s026.docx]

**S26 Table.** Inhibitory effect of varying concentrations of NSC177383 on the enzymatic activity of DiMT protein.

| **NSC177383 (µM)** | **0** | **40** | **80** | **120** | **140** | **180** |
| --- | --- | --- | --- | --- | --- | --- |
| **Mean Percent Inhibition** | 0 | 17.3 | 30.5 | 45.9 | 50.6 | 63.6 |
|  | 0 | 18.1 | 30.7 | 41.2 | 45.4 | 62.4 |
|  | 0 | 14.9 | 34.4 | 40.2 | 42.2 | 64.2 |
| **Average** | 0 | **16.8** | **31.9** | **42.4** | **46.1** | **63.4** |
| **SEM** | **0** | **0.8** | **1.0** | **1.4** | **2.0** | **0.4** |
