# Supplementary material for: Characterization of a unique catechol-O-methyltransferase as a molecular drug target in parasitic filarial nematodes
Source: PLoS Negl Trop Dis. 2024 Aug 30;18(8):e0012473. doi: 10.1371/journal.pntd.0012473 (PMC11392244; doi:10.1371/journal.pntd.0012473)
Supplement: S27 Table — (DOCX) [file pntd.0012473.s027.docx]

**S27 Table.** Inhibitory effect of varying concentrations of NSC145612 on the enzymatic activity of DiMT protein.

| **NSC145612 (µM)** | **0** | **50** | **100** | **150** | **200** | **250** |
| --- | --- | --- | --- | --- | --- | --- |
| **Mean Percent Inhibition** | 0 | 68.4 | 57.6 | 66.2 | 77.2 | 84.0 |
|  | 0 | 38.5 | 50.0 | 62.4 | 75.4 | 87.6 |
|  | 0 | 36.2 | 61.2 | 82.9 | 75.6 | 81.7 |
| **Average** | **0** | **47.7** | **56.3** | **70.5** | **76.1** | **84.4** |
| **SEM** | **0** | **8.5** | **2.7** | **5.2** | **0.5** | **1.4** |
