# Supplementary material for: Characterization of a unique catechol-O-methyltransferase as a molecular drug target in parasitic filarial nematodes
Source: PLoS Negl Trop Dis. 2024 Aug 30;18(8):e0012473. doi: 10.1371/journal.pntd.0012473 (PMC11392244; doi:10.1371/journal.pntd.0012473)
Supplement: S28 Table — (DOCX) [file pntd.0012473.s028.docx]

**S28 Table.** Inhibitory effect of varying concentrations of NSC133100 on the enzymatic activity of DiMT protein.

| **NSC133100 (µM)** | **100** | **200** | **300** | **400** | **500** |
| --- | --- | --- | --- | --- | --- |
| **Mean Percent Inhibition** | 24.8 | 32.0 | 33.9 | 40.4 | 51.5 |
|  | 27.0 | 34.7 | 41.0 | 46.0 | 54.8 |
|  | 28.5 | 35.8 | 42.1 | 46.0 | 55.7 |
| **Average** | **26.8** | **34.1** | **39.0** | **44.1** | **54.0** |
| **SEM** | **0.9** | **0.9** | **2.1** | **1.5** | **1.0** |
