# Supplementary material for: Characterization of a unique catechol-O-methyltransferase as a molecular drug target in parasitic filarial nematodes
Source: PLoS Negl Trop Dis. 2024 Aug 30;18(8):e0012473. doi: 10.1371/journal.pntd.0012473 (PMC11392244; doi:10.1371/journal.pntd.0012473)
Supplement: S29 Table — (DOCX) [file pntd.0012473.s029.docx]

**S29 Table.** Inhibitory effect of varying concentrations of NSC56410 on the enzymatic activity of DiMT protein.

| **NSC56410 (µM)** | **50** | **100** | **150** | **200** | **250** | **300** | **350** | **400** |
| --- | --- | --- | --- | --- | --- | --- | --- | --- |
| **Mean Percent Inhibition** | 18.0 | 26.6 | 31.0 | 34.6 | 41.2 | 48.3 | 53.8 | 62.5 |
|  | 26.7 | 33.3 | 35.0 | 41.9 | 38.8 | 48.6 | 59.3 | 62.5 |
|  | 20.5 | 21.3 | 25.0 | 31.9 | 51.8 | 46.0 | 53.7 | 63.8 |
| **Average** | **21.7** | **27.1** | **30.3** | **36.1** | **43.9** | **47.6** | **55.6** | **62.9** |
| **SEM** | **2.1** | **2.8** | **2.4** | **2.4** | **3.3** | **0.7** | **1.5** | **0.4** |
