# Supplementary material for: Characterization of a unique catechol-O-methyltransferase as a molecular drug target in parasitic filarial nematodes
Source: PLoS Negl Trop Dis. 2024 Aug 30;18(8):e0012473. doi: 10.1371/journal.pntd.0012473 (PMC11392244; doi:10.1371/journal.pntd.0012473)
Supplement: S30 Table — (DOCX) [file pntd.0012473.s030.docx]

| **NSC227186 (µM)** | **10** | **40** | **70** | **100** | **130** | **160** |
| --- | --- | --- | --- | --- | --- | --- |
| **Mean Percent Inhibition** | 25.0 | 40.1 | 40.5 | 44.8 | 46.6 | 50.6 |
|  | 23.9 | 37.1 | 43.1 | 43.5 | 49.3 | 48.4 |
|  | 30.2 | 38.3 | 39.6 | 45.8 | 48.1 | 57.2 |
| **Average** | **26.4** | **38.5** | **41.1** | **44.7** | **48.0** | **52.0** |
| **SEM** | **1.6** | **0.7** | **0.8** | **0.5** | **0.6** | **2.2** |

**S30 Table.** Inhibitory effect of varying concentrations of NSC227186 on the enzymatic activity of DiMT protein.
