# Supplementary material for: Characterization of a unique catechol-O-methyltransferase as a molecular drug target in parasitic filarial nematodes
Source: PLoS Negl Trop Dis. 2024 Aug 30;18(8):e0012473. doi: 10.1371/journal.pntd.0012473 (PMC11392244; doi:10.1371/journal.pntd.0012473)
Supplement: S1 Fig — (A) Plotting colors represent phi-psi backbone conformational areas: red represents the most favored regions, brown and yellow represent additional and generously allowed regions, while light-yellow patches represent regions that are not allowed. The sky-blue dots represent (φ, ψ) angles for each residue of the predicted structure. The α-helical (around 0, -45° and -75°, -45°) and β-sheet (near 135° and 180°) regions are highlighted with red color. Majority of the blue dots amino acid residues lie within the β-sheet and right-handed α-helix regions. (B) The Ramachandran plot statistics reveal that 92.7% of residues lie within the most favored region, indicating the reliability of the predicted model. (PPTX) [file pntd.0012473.s031.pptx]

## Slide 1
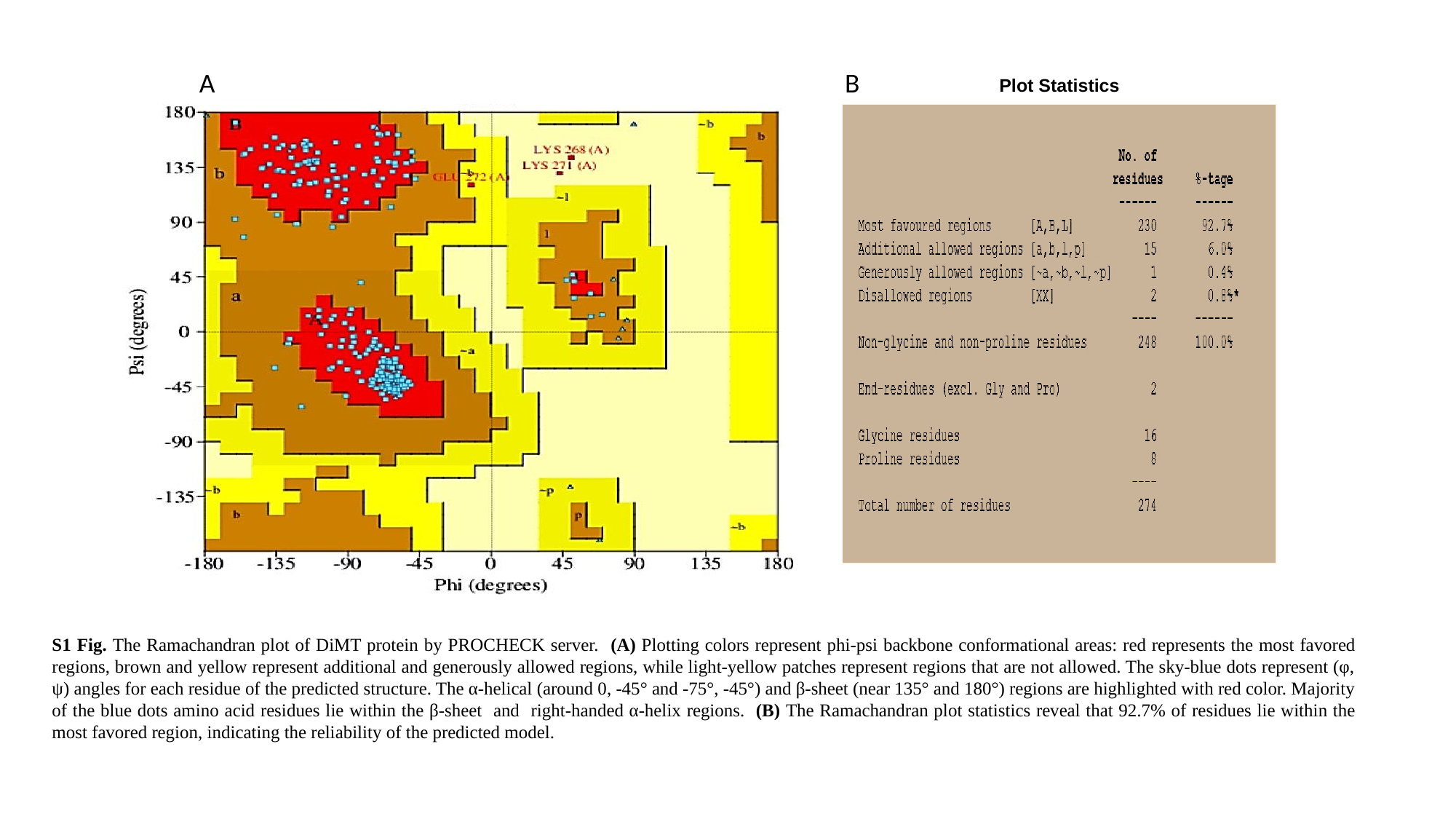

A
B
Plot Statistics
S1 Fig. The Ramachandran plot of DiMT protein by PROCHECK server. (A) Plotting colors represent phi-psi backbone conformational areas: red represents the most favored regions, brown and yellow represent additional and generously allowed regions, while light-yellow patches represent regions that are not allowed. The sky-blue dots represent (φ, ψ) angles for each residue of the predicted structure. The α-helical (around 0, -45° and -75°, -45°) and β-sheet (near 135° and 180°) regions are highlighted with red color. Majority of the blue dots amino acid residues lie within the β-sheet and right-handed α-helix regions.  (B) The Ramachandran plot statistics reveal that 92.7% of residues lie within the most favored region, indicating the reliability of the predicted model.
