# Supplementary material for: Characterization of a unique catechol-O-methyltransferase as a molecular drug target in parasitic filarial nematodes
Source: PLoS Negl Trop Dis. 2024 Aug 30;18(8):e0012473. doi: 10.1371/journal.pntd.0012473 (PMC11392244; doi:10.1371/journal.pntd.0012473)
Supplement: S2 Fig — (A) PDBsum-predicted secondary structural topology of human COMT (PDB code: 3BWM). 3-D structure of human COMT (right side) displaying the arrangement of β-strands (order 3214576) and the overall fold of the enzyme. (B) DiMT protein’s structural topology and 3-D structure displaying similar number of β-strands and arrangement of the overall fold of the human COMT protein structure. PyMoL (https://www.pymol.org) was used to visualize β-strands within the 3-D structure of COMT and DiMT. (PPTX) [file pntd.0012473.s032.pptx]

## Slide 1
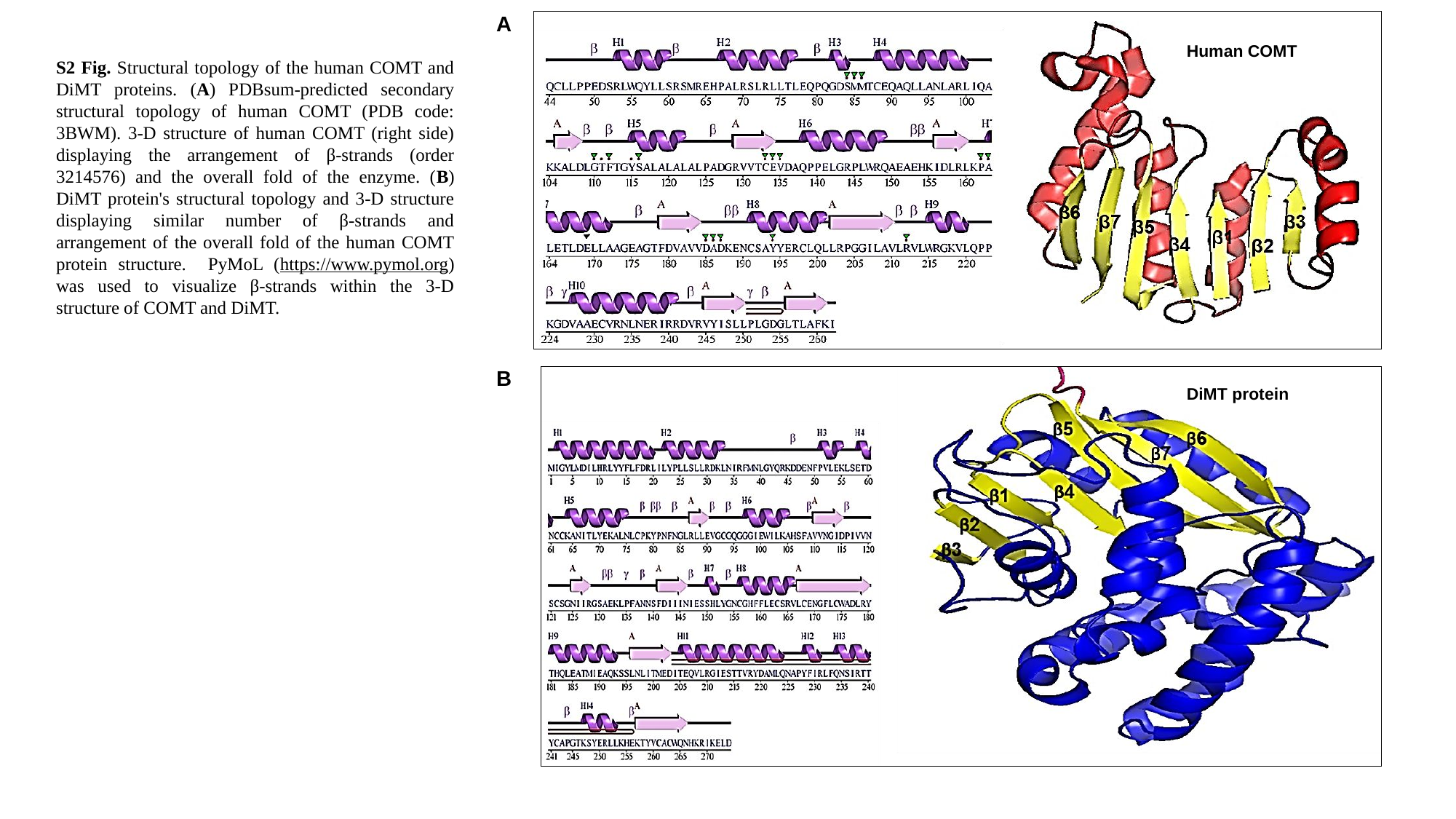

A
Human COMT
S2 Fig. Structural topology of the human COMT and DiMT proteins. (A) PDBsum-predicted secondary structural topology of human COMT (PDB code: 3BWM). 3-D structure of human COMT (right side) displaying the arrangement of β-strands (order 3214576) and the overall fold of the enzyme. (B) DiMT protein's structural topology and 3-D structure displaying similar number of β-strands and arrangement of the overall fold of the human COMT protein structure. PyMoL (https://www.pymol.org) was used to visualize β-strands within the 3-D structure of COMT and DiMT.
B
DiMT protein
