# Supplementary material for: Characterization of a unique catechol-O-methyltransferase as a molecular drug target in parasitic filarial nematodes
Source: PLoS Negl Trop Dis. 2024 Aug 30;18(8):e0012473. doi: 10.1371/journal.pntd.0012473 (PMC11392244; doi:10.1371/journal.pntd.0012473)
Supplement: S3 Fig — (PPTX) [file pntd.0012473.s033.pptx]

## Slide 1
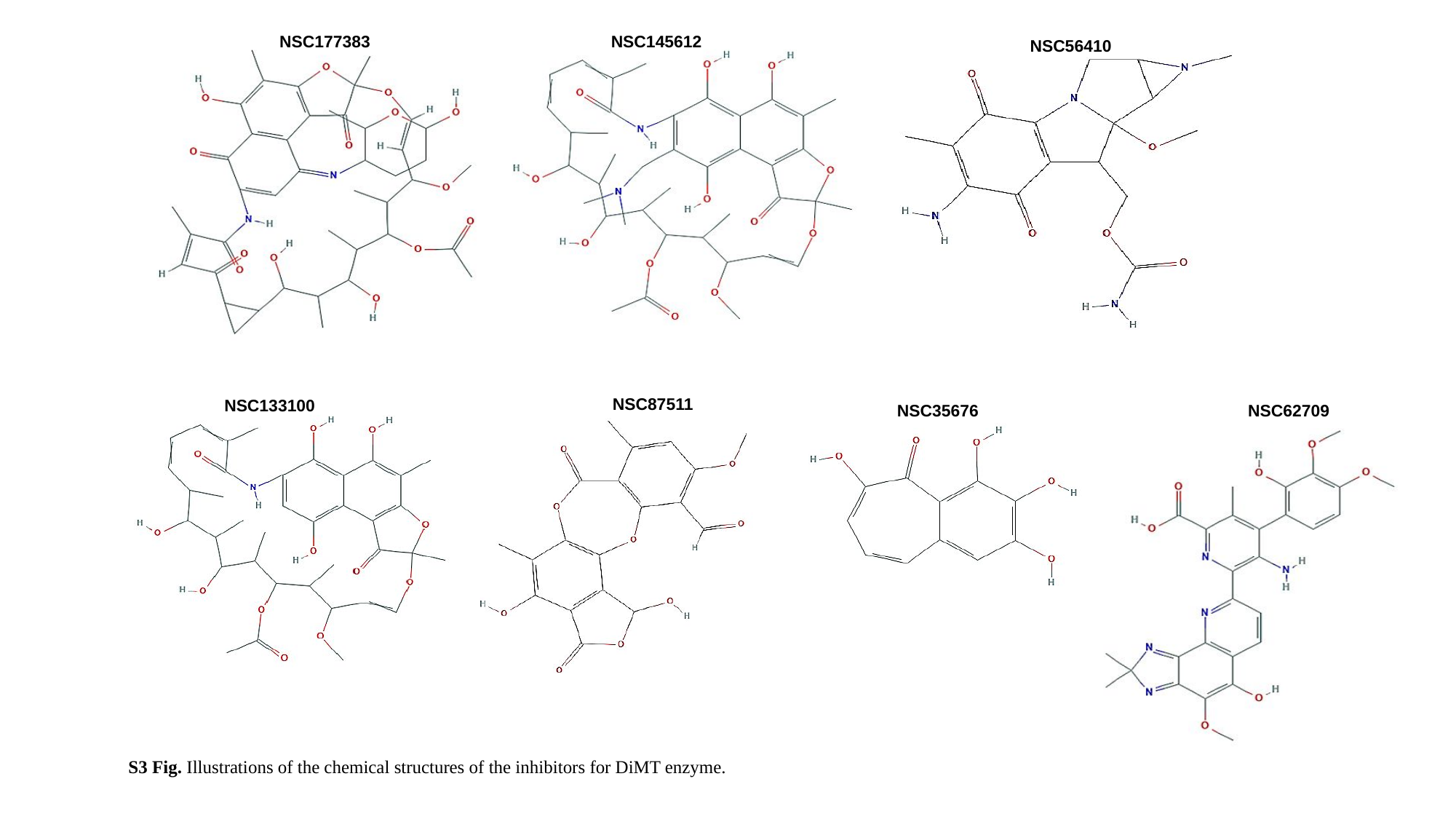

A
NSC177383
NSC145612
NSC56410
NSC87511
NSC133100
NSC35676
NSC62709
S3 Fig. Illustrations of the chemical structures of the inhibitors for DiMT enzyme.
